# Tipping the balance between erythroid cell differentiation and induction of anemia in response to the inflammatory pathology associated with chronic trypanosome infections

**DOI:** 10.3389/fimmu.2022.1051647

**Published:** 2022-11-07

**Authors:** Hang Thi Thu Nguyen, Magdalena Radwanska, Stefan Magez

**Affiliations:** ^1^ Department of Biochemistry and Microbiology, Ghent University, Ghent, Belgium; ^2^ Laboratory for Biomedical Research, Ghent University Global Campus, Incheon, South Korea; ^3^ Laboratory of Cellular and Molecular Immunology, Vrije Universiteit Brussel, Brussels, Belgium; ^4^ Department of Biomedical Molecular Biology, Ghent University, Ghent, Belgium

**Keywords:** Trypanosomosis, tissue resident macrophages, extramedullary erythropoiesis, immunopathology, lactic acidosis

## Abstract

Infection caused by extracellular single-celled trypanosomes triggers a lethal chronic wasting disease in livestock and game animals. Through screening of 10 *Trypanosoma evansi* field isolates, exhibiting different levels of virulence in mice, the current study identifies an experimental disease model in which infection can last well over 100 days, mimicking the major features of chronic animal trypanosomosis. In this model, despite the well-controlled parasitemia, infection is hallmarked by severe trypanosomosis-associated pathology. An in-depth scRNA-seq analysis of the latter revealed the complexity of the spleen macrophage activation status, highlighting the crucial role of tissue resident macrophages (TRMs) in regulating splenic extramedullary erythropoiesis. These new data show that in the field of experimental trypanosomosis, macrophage activation profiles have so far been oversimplified into a bi-polar paradigm (M1 vs M2). Interestingly, TRMs exert a double-sided effect on erythroid cells. On one hand, these cells express an erythrophagocytosis associated signature. On another hand, TRMs show high levels of *Vcam1* expression, known to support their interaction with hematopoietic stem and progenitor cells (HSPCs). During chronic infection, the latter exhibit upregulated expression of *Klf1*, *E2f8*, and *Gfi1b* genes, involved in erythroid differentiation and extramedullary erythropoiesis. This process gives rise to differentiation of stem cells to BFU-e/CFU-e, Pro E, and Baso E subpopulations. However, infection truncates progressing differentiation at the orthochromatic erythrocytes level, as demonstrated by scRNAseq and flow cytometry. As such, these cells are unable to pass to the reticulocyte stage, resulting in reduced number of mature circulating RBCs and the occurrence of chronic anemia. The physiological consequence of these events is the prolonged poor delivery of oxygen to various tissues, triggering lactic acid acidosis and the catabolic breakdown of muscle tissue, reminiscent of the wasting syndrome that is characteristic for the lethal stage of animal trypanosomosis.

## Introduction

The extracellular protozoan parasite *Trypanosoma evansi* (*T. evansi*), also referred to as *Trypanosoma brucei evansi (T. b. evansi)* (1), is the causative agent of the animal disease “Surra”, as well as rare cases of atypical human trypanosomosis (2–4). *T. evansi* is not relying on tsetse transmission only, but uses a wide range of insect vectors and even vampire bats (5). Hence, this parasite has been able to move out of Africa, and is present in South America, vast areas of Asia, and parts of Oceania (5, 6), and occasionally crops up in Europe as a result of uncontrolled import of infected animals (7). The host reservoir of *T. evansi* includes livestock animals such as cattle, horses, camels, pigs, goats, and water buffalos, as well as companion animals such as dogs, and game animals (8). The ability to colonize a diverse host range implies that *T. evansi* has the ability to very efficiently evade the mammalian immune system. It is generally accepted that the process of antigenic variation of the Variant Surface Glycoprotein (VSG) coat is a key factor here (9). In addition, recent data that was obtained by us at single-cell resolution, shows that *T. evansi* undermines the host immune system by (i) reducing early B cell linage lymphopoiesis in bone marrow, (ii) inducing low-functionality B cell differentiation while preventing naïve B cells replenishment of the periphery, and (iii) abrogating B cell memory induction. *T. evansi* also triggers early-stage IgG immunoglobulin isotype switching, allowing to escape from natural IgM antibody-mediated trypanocidal activity (10).

*T. evansi* parasites exhibit variable levels of virulence, depending on the host and geographical origin (8, 11, 12). In experimental models, trypanosomosis virulence patterns are typically described in terms of peak parasitemia height, anemia development or weight loss, tissue tropism and host survival time (12). In livestock animals, *T. evansi* often triggers a chronic wasting disease, for which the underlying immunological mechanisms have not been thoroughly investigated (5). Hence, having access to a collection of *T. evansi* isolates, exhibiting different levels of virulence, offers a unique approach to investigate the driving mechanisms of the detrimental wasting disease outcome of animal trypanosomosis (AT). Addressing this at the level of single-cell resolution offers new insights into the trypanosome-host biology that have not been revealed before.

So far, conventional immunological studies combined with bulk-RNA sequencing, have suggested that while antibody response are important for trypanosome parasitemia control (13), it is the balance of pro- and anti-inflammatory cytokines production, accompanied by activation and alteration of classical and alternatively activated macrophages populations, that determines the chronicity of AT infections (14–18). In general, the early stage of trypanosomosis is hallmarked by the induction of a Th1 T cell immune response and the presence of pro-inflammatory cytokines such as INFγ, TNFα, and IL-1β (19–21). These cytokines stimulate classically activated pro-inflammatory macrophages, so-called M1 macrophages. These in turn contribute to the control of parasitemia through TNF and Nitric Oxide production and/or phagocytosis (22–24). However, maintaining highly activated M1 cells can lead to infection-induced pathological features such as systemic immune response syndrome or severe anemia. In contrast, the chronic stage of infection in trypanotolerant animals, so far only described for *T. congolense* infections, is hallmarked by the presence of a Th2 immune environment, with anti-inflammatory cytokines such as IL-10, IL-4, and TGFβ (25). These cytokines, especially IL-10, induce the expression and activation of arginase in alternatively activated M2 macrophages. The switch from M1 to M2 cells has been suggested to be important for dampening prolonged infection-associated inflammation (26, 27). The crucial role of IL-10 in the regulation of trypanosomosis associated pathology has been well documented using IL-10^-/-^ mice (26, 28). Over the last decade, various results obtained in experimental immunology models gave rise to the concept that alteration of the M1/M2 macrophage balance, and production of their related cytokines, governs the outcome of disease. However, recent studies suggest that the bipolar paradigm might not be fully applicable *in vivo*, where macrophages can be more heterogeneous (29, 30). This is especially true when taking into account the presence of tissue-resident macrophage populations. Hence, to grasp the importance of M1/M2 macrophage biology in chronic trypanosomosis, the *T. evansi* context offers a unique model system.

Chronic livestock AT is characterized by the occurrence of a wasting syndrome that drives the fatal outcome of infection, hallmarked by persistent severe anemia. The latter has been characterized as ‘anemia of chronic inflammation/disease’ (ACD), involving factors such as galectin-3, a driver of erythrophagocytosis, and the macrophage migration inhibitory factor MIF responsible for iron retention by the mononuclear phagocyte system (31, 32). MIF also promotes M1 macrophage polarization (18). TNF, originally identified as cachectin, as well as the macrophage activator IFNγ, have also been described as a key player in trypanosomosis-induced anemia (33, 34). As anemia results in tissue hypoxia, a compensatory stress-induced erythropoiesis can be triggered. When occurring in the spleen or even liver, this process is referred to as extramedullary erythropoiesis (35). At the level of the spleen, it is initiated by either the migration of bone marrow erythroid progenitors into spleen or by the expansion of splenic resident erythroid progenitors (36, 37). Subsequently, these progenitors pass through several differentiation stages, during which cells gain fully mature features of reticulocytes. This is achieved by the removal of their nucleus and other organelles, as well as gaining flexibility by membrane-cytoskeleton rearrangement in the final steps of red blood cell formation (38). Interestingly, the occurrence of spleen extramedullary erythropoiesis has been described in various experimental trypanosomosis models (32, 39). However, as infection-induced anemia is a key feature of trypanosomosis in general, this rescue mechanism is clearly not able to compensate for ongoing accelerated erythrophagocytosis. So far, no in-depth mechanisms have been described that can explain the failure of extramedullary erythropoiesis to compensate for infection-associated erythrophagocytosis. Hence, having access to a chronic experimental *T. evansi* model that mirrors the wasting disease pathology observed in livestock trypanosomosis, has now allowed us to (i) investigate the roles of antibodies and cytokines, (ii) address macrophage activation status at a single-cell level, and (iii) analyze the reticulocyte differentiation process during extramedullary erythropoiesis. Our results show that chronic *T. evansi* infection is characterized by the presence of high circulating antibody titers and low parasite numbers. At the same time, progressing splenic tissue pathology is hallmarked by the expansion of differentiating myeloid cell populations and erythroid progenitors. However, the final arrest of splenic erythrocytes differentiation at the level of the orthochromatic erythrocyte stage, is a major hurdle that prevents the maintenance of adequate circulating red blood cells. Consequently, chronic *T. evansi* trypanosomosis results in the development of severe systemic lactic acid acidosis, and a lethal wasting syndrome that is observed in all livestock trypanosome infections, characterized by severe muscular dystrophy.

## Material and methods

### Mice and ethics statement

7-9 weeks-old female C57Bl/6 mice were supplied by KOATECH (Gyeonggi-do, Korea), hosted in individually Ventilated Cages (IVC) and provided with cage enrichment. The Ghent University Global Campus Institutional Animal Care (GUGC IACUC) approved experimental animal protocols (IACUC 2019-003/016/023, 2020-004/012/016, 2021-019, and 2022-006).

### Parasites

*T. evansi Vietnam* (STOCK2) (ITMAS 180697), *T. evansi* STIB816 (ITMAS 140799A), *T. evansi* Rotat 1.2 (ITMAS 020298), *T. evansi Colombia* (E9.C12) (ITMAS 150799), *T. evansi* E18 (ITMAS 140799B)*, T. evansi* Merzouga 93 (ITMAS 150399C), *T. evansi* Zagoura III-25 (ITMAS 120399C), *T. evansi* KAZACHSTAN (ITMAS 060297), *T. evansi* KETRI 2479 (ITMAS 100883A), *T. evansi* 2840 (ITMAS 110297) were obtained from the Institute for Tropical Medicine, Antwerp, Belgium.

### Parasites gDNA extraction and PCR for RoTat 1.2 gene detection

Total genomic DNA of parasite isolates was extracted from infected mouse blood at the level of approximately 2x10^7^ parasites/mL, using a DNeasy Blood & Tissue Kit (Qiagen, Germany) according to the manufacturer’s instructions. The PCR was carried out as previously described (40), with the following changes: the amount of extracted genomic DNA used as starting material (250 ng instead of 3000 ng) and the addition of 10% DMSO to the reaction mixture.

### Infection

Mice were infected by intraperitoneal (i.p) injection of with 200 parasites in 100 μL of Dulbecco’s Phosphate Buffered Saline (DPBS; Invitrogen, CA, USA). Parasitemia and red blood cell number were quantified in 2.5 µL blood, taken from the tail vein, using a hemocytometer and a light microscope, after the blood was diluted (1/200) in Dulbecco’s Phosphate Buffered Saline (DPBS; Invitrogen, CA, USA). Infections were followed till a point where mice were euthanized in accordance with the IACUC guidelines, taking into account overall infection-induced morbidity, locomotor activity, anemia and/or hyper-parasitemia, and weight loss. Overall body weight loss was determined post-mortem, after removal of spleen and liver in order to eliminate the effects of infection-induced hyper spleno-hepathomegaly.

### Virulence classification

Infected mice were CO_2_ euthanized when they reached the human end point specified in ethical approval documents. The mean survival time (MST) of mice infected with the same *T. evansi* isolate was used to classify the parasites’ virulence. The Wilcoxon and Log-rank tests were used to classify results.

### Cell isolation and flow cytometry analysis

Spleens and bone marrow cells from naïve and infected mice were isolated at day 7, 14, 28, 42 p.i. for *T. evansi* Merzouga 93. Time points were selected based on the survival time of infected mice and their first parasitemia peak occurrence. Single cell suspensions were prepared by homogenizing spleen in 6 mL of DMEM (Capricorn Scientific, Ebsdorfergrund, Germany) supplemented with 10% FBS (Atlas Biologicals, CO, USA) and 1% penicillin/streptomycin using gentleMACS Dissociator (Miltenyi Biotec, Bergisch Gladbach, Germany). Cells were spun down at 314 x G for 7 minutes at 4°C after being filtered through a 70 µm pore size cell strainer (SPL Life Sciences, Gyeongi-do, Korea). Prior to flow cytometry analysis, non-specific binding was blocked by incubating cells with anti-CD16/32 antibody (Biolegend CA, USA) (1:1000 dilution) at 4°C for 30 minutes in the dark. Afterwards, 10^5^ cells per sample were incubated in the dark at 4°C for 30 minutes with a mixture of antibodies specific for distinct cells populations, followed by flow cytometry analysis with a BD AccuriTM C6 Plus flow cytometer (BD Biosciences, CA, USA). Anti-Ter119 PE and anti-CD71 FITC were purchased from Biolegend (CA, USA) and used at a 1/600 dilution. The total number of cells in each population was determined by multiplying the percentages of subsets within a series of marker negative or positive gates, with the total live cell number determined for each cell preparation in combination with microscopy live cell counts for every individual cell preparation.

### Parasite isolation and VSG preparation

Heparinized blood was obtained from a *T. evansi* infected mouse when parasitemia reached approximately 5 x 10^7^ parasites/ml (before the first parasitemia peak). Parasites were separated from red blood cells by ion-exchange DEAE-cellulose (DE52) column chromatography (Whatman, Maidstone, UK). Separated parasites were then collected in phosphate saline glucose buffer (44 mM NaCl, 57 mM Na_2_HPO_4_, 3 mM NaH_2_PO_4_, 55 mM glucose) before being washed twice with DPBS (Invitrogen, Carlsbad, CA) by centrifuging at 1500 x G for 7 minutes, followed by 30 minutes incubation on ice in 1mL Baltz buffer (0.125 M phosphate buffer pH 5.5 containing 1% Glucose) and 5 minutes incubation at 37°C. Soluble VSG (sVSG) present in the supernatant was collected after 10 minutes centrifugation of the sample at 15000 x g. The concentration of sVSG was then estimated using the Bradford protein assay kit (Bio-rad, CA, USA), before being frozen at -20°C (41).

### Measurement of total and anti-VSG antibodies titers in plasma samples

Fifty μL sVSG/well was used to coat Half Area Clear Flat Bottom Polystyrene High Bind Microplate (Corning, NY, USA) at 4°C overnight, at a concentration of 4 μg/mL. Plasma was obtained from infected mice on the same day as the spleen. DPBS (Invitrogen, Carlsbad, CA) was used to generate a 1:2 serial dilution series of plasma samples, starting from 1:100 to 1:204800. IgM, IgG2b, IgG2c, and IgG3 antibody titers were measured using horseradish peroxidase-labeled anti-IgM, anti-IgG2b, anti-IgG2c, and anti-IgG3 antibodies (Southern Biotech, Alabama, USA). To access total plasma antibodies titers, 5 μg/mL of Goat Anti-Mouse Ig (Southern Biotech) was coated overnight in 96 wells Immuno maxi-binding plate (SPL Life Sciences, Gyeonggi-do, Korea) at 4°C. Plasma samples - the same as used for anti-VSG antibodies titers determination - were subsequently processed according to the same protocol outlined above.

### Quantification of cytokines

ELISA MAX™ Deluxe Set Mouse INFɣ, TNFα and IL10 sets were used to assess the concentrations of INFɣ, TNFα and IL10 cytokines in plasma (Biolegend, CA, USA). In brief, plasma were taken from infected mice at various time points. In brief, plasma samples were collected from infected mice at different time points. Subsequently, different samples (non-diluted, 1:10 dilution, 1:100 dilution) were used to estimate concentrations of INFɣ, TNFα and IL10 following the manufacturers’ protocol.

### Single cell RNA sequencing

Spleen single cell suspensions were isolated from mice infected with *T. evansi* Merzouga 93 at 3 different time points: Naïve, 14 dpi, and 42 dpi. To assess the effect of pooling samples on scRNA-seq data analysis, spleen cells suspension from a single mouse and pooled spleen cells suspension from 3 individual mice (1:1:1 ratio) were prepared at 42 dpi. This approach enabled to compare the reproducibility of the results obtained from single and pooled samples after scRNA-seq. Cell libraries were prepared using Chromium Single Cell 3’ Reagent User Kit (v3 chemistry) provided by 10X Genomics. Sequencing was conducted on a NovaSeq600 platform using TruSeq Illumina primers. Raw based call (BCL) files generated from sequencer were processed using Cellranger pipeline provided by 10X Genomics.

### Single cell RNA sequencing analysis

BCL files were demultiplexed into sample specific FASTQ files using *cellranger mkfastq*. Using *cellranger count* command, sequenced reads were aligned to the 10X prebuilt mouse reference genome package (mm10) using STAR aligner. Subsequently, cell barcodes and Unique Molecular Identifiers were counted, filtered, and corrected to generate expression matrices for each sample, which were used for further data processing and analysis. Dataset of each sample were adjusted to remove ambient RNAs contamination using SoupX (42). Also, genes which are known for causing technical background noise, Gm42418 and AY036118 (43), were removed from expression matrices prior to Seurat (v4.0) processing and downstream analysis (44). Standard procedure for filtering low quality cells were applied for each sample separately. In brief, cells having less than 100 genes or more than 6000 genes, cells expressing mitochondrial genes counting for more than 10% of total genes detected were excluded from analysis. To evaluate the similarity between data generated from an individual mouse and pooled 3 mice, 42dpi single and 42dpi pool datasets were merged for dimensionally reduction and clustering analysis without applying batch effects removal methods. For combined total spleen cells dataset and subsequently, total macrophages dataset, all samples were merged, and batch effects were removed by Harmony package (45) to create a combined dataset for all samples. Harmony corrected embedding was used to perform normalization, dimensionally reduction, and clustering. Cell type annotation for Macrophages, Hematopoietic stem and progenitor cells, and Erythroid were carried out using gene markers collected from literature. Intercellular communication analysis was done for combined dataset of all spleen cells from all time points using Cellchat package (46) to predict the interaction between cell populations based on their ligands and receptors expression.

Clusters expressing Macrophages markers were subsetted and re-performed dimensionally reduction and clustering to generate a combined Macrophages dataset for all samples. Similarly, clusters expressing HSPC and Erythrocytes markers were subsetted for generating Erythrocytes lineage dataset. The sub-populations of these datasets were annotated using markers collected from the literature and SingleR package. To obtain differentially expressed genes among annotated sub-populations, the Wilcoxon rank sum test was performed with the FindMarkers function of Seurat. All *p values* were corrected for multiple comparisons using the Benjamin-Hochberg method. For gene set enrichment analysis, we used clusterProfiler package to perform pre-ranked gene set enrichment analysis on 50 hallmark gene sets of MSigDB collection (47). We used average log2 fold change detected from FindMarkers function as gene score to rank genes. Enrichment plots for specific pathways were obtained using the enrichplot package (48). Genes included in HALLMARK_INTERFERON_GAMMA_RESPONSE, HALLMARK_TNFA_SIGNALING_VIA_NFKB, HALLMARK_INFLAMMATORY_RESPONSE pathways of MSigBD hallmark gene sets collection were used to calculate Interferon gamma response, TNF alpha signaling, and Inflammatory response scores respectively. BBrowser3 (BioTuring Inc., San Diego California USA) was used to visualize the Seurat object for UMAP and gene expression.

### Statistical analysis

Graphpad prism statistical software (GraphPad Software Inc. San Diego, CA) was used to perform Student’s t-test or one-way ANOVA tests as indicated in the result section. Data were presented as Mean + SD unless stated otherwise. Values of P ≤ 0.05 are considered statistically significant.

## Result

### Classification of 10 *T. evansi* isolates into a spectrum of virulent categories

**Figure 1:**
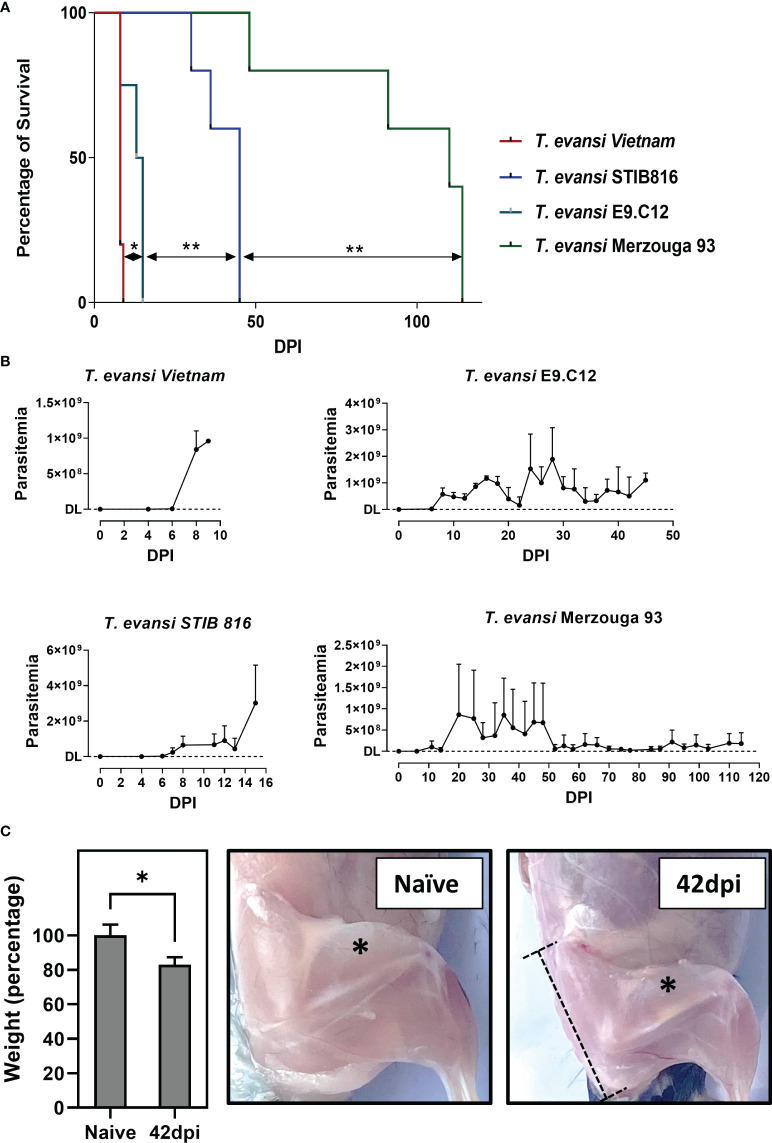
Chronic infection by *T. evansi* Merzouga 93 is similar to infections in domestic animals. **(A)** Survival curve of 4 representative isolates: *T. evansi* Vietnam (hyper-virulent), *T. evansi* STIB816 (virulent), *T. evansi* E9.C12 (intermediate), and *T. evansi* Merzouga 93 (chronic). *p≤ 0.05, **p≤ 0.01 by Log-rank test. **(B)** Parasitemia profiles of 4 isolates during infection. Data are presented as mean + SD from 5 individual mice per group. The Y axis indicates the number of parasites, DL: detection limit (4x10^5^ parasites/ml blood). **(C)** Left panel: Weight reduction in percentage of infected mice (42dpi) relative to naïve mice. Data is presented as mean + SD of mice’s body weight after subtracting the weight of spleen and liver. *p ≤ 0.05 by Student t-test vs naïve values. Right panels: Catabolic muscle breakdown observed during chronic infection of *T. evansi* Merzouga 93. The asterisk indicates the location of quadriceps muscle, the dashed bar indicates the location of the Pelvis bone.

*T. evansi* parasites used in this study originate from 8 different countries and distinct hosts, making up for 10 non-cloned field samples, including 9 Type A isolates that are most common around the world, and one East-African *T. evansi* type B isolate (**Table 1**; **Figure S1**). These isolates are categorized into 4 different virulence groups, based on mean survival time (MST) of mice, after challenge with the same infection dose (200 parasite/mouse): (i) hyper-virulent; MST shorter than 10 days, (ii) virulent; MST 11-30 days, (iii) Intermediate; MST 30-60 days, and (iv) chronic; MST more than 60 days. Mice infected with distinct isolates in the same group show non-significant differences in MST, while combined survival data of each group are significantly different from each other when tested by Log-rank and Wilcox tests (**Table S1**). Of the ten isolates tested, *T. evansi* Merzouga 93 infection exhibits the longest surviving period in mice, reaching a MST of 110 days, with occasional infections lasting for up to 8 months. From each category outlined above, 4 representative isolates were selected for further investigation, i.e. the Vietnam isolate, the STIB816 isolate, the E9.C12 (Colombia) isolate, and the Merzouga 93 isolate. Survival curves and parasitemia profiles (**Figures 1A, B**) show that the 4 isolates developed parasitemia profiles with varying numbers of peaks. In mice infected with hyper-virulent isolate, the parasitemia pattern results in only one single peak, reaching extreme levels of over 1x 10^9^ parasites/ml. A similar overall pattern is seen in mice infected with the virulent STIB816 isolate, however here infection lasts over two weeks. Infections caused by intermediate E9.C12 isolate resulted in multiple high parasitemia peaks, without the occurrence of any visible infection-associated pathology, over a period of up to 5 weeks. For all three groups, mice were euthanized when hyper-parasitemia, coinciding with acute locomotor dysfunction, resulted in visible morbidity. The chronic Merzouga 93 isolate is characterized by several early-stage parasitemia waves, followed by a prolonged chronic infection with low well-controlled parasite numbers in the circulation (**Figure 1B**). In this case however, the infection caused a wasting syndrome, hallmarked by a significant decrease in body mass by day 42 post infection (**Figure 1C**, left panel). Comparing the muscle mass of both quadriceps and gastrocnemius muscles clearly shows the catabolic muscle breakdown observed during the chronic infection stage. Hence, pelvis and femur bones of infected mice (42 dpi) are more visible due to muscular atrophy, whereas they are neatly covered by muscle tissue in naïve mice (**Figure 1C**, right panels). It is important to mention that livestock animals suffering from AT usually do not succumb to high parasitemia, and hence mouse trypanosome models that are hallmark by uncontrolled parasitemia might not be the best to gain profound insights into the disease mechanism that drive AT. Hence, as *T. evansi* Merzouga 93 infections are characterized by a long survival period, accompanied by well-controlled parasitemia peaks, as well as a catabolic muscle disorder, resembling livestock AT pathology, this isolate was chosen for further investigation.

**Table 1:** Information of 10 *T. evansi* isolates and their classification based on mean survival time of infected mice.

Classification	Strains	Country	Host	lso. Year	MST (days)	Type
**Hyper-virulent**	Kazakhstan	Kazakhstan	Camel	1995	7	A
	Vietnam	Vietnam	Buffalo	1996	8	A
	**Group mean ± SD**				**7.5 ± 0.71**	
**Virulent**	STIB 816	China	Camel	1978	14	A
	RoTat 1.2	Indonesia	Buffalo	1982	12	A
	CAN 86	Brazil	Dog	1986	17	A
	**Group mean ± SD**				**14.33 ± 2.5**	
**Intermediate**	Ketri 2479	Kenya	Camel	1981	45	B
	Ketri 2480	Kenya	Camel	1981	35	A
	E9.C12	Colombia	Horse	1973	45	A
	Zagoura III-25	Morocco	Camel	1998	40	A
	**Group mean ± SD**				**41 ± 4.78**	
**Chronic**	Merzouga 93	Morocco	Camel	1998	110	A
	**Group mean ± SD**				***NA***	

In bold: mean survival time ± SD of mice with in same virulence group. NA, not applicable.

### An anti-inflammatory cytokine balance hallmarks chronic infection

**Figure 2:**
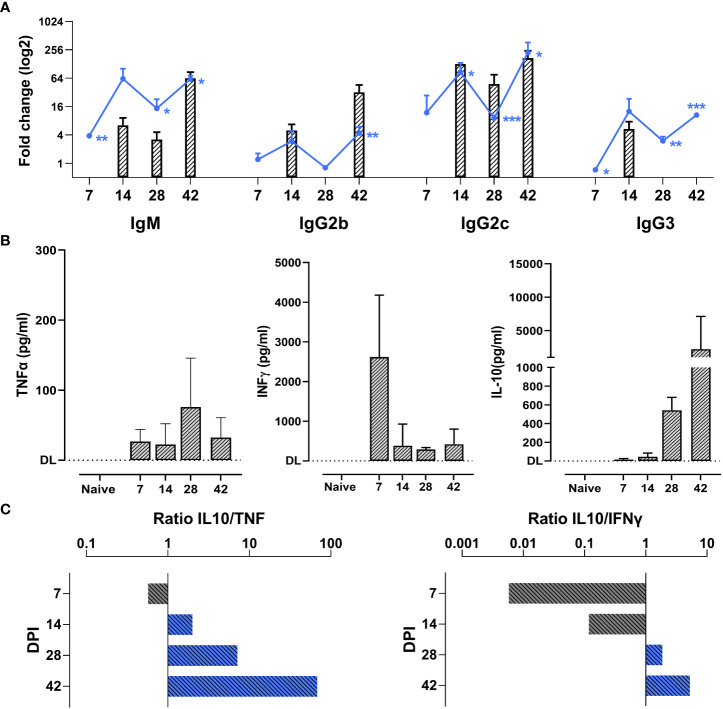
Chronic *T. evansi* Merzouga 93 infection hallmarked by high antibody titers and anti-inflammatory cytokine response. **(A)** Total antibodies titers (line graphs) and anti-VSG antibodies titers (bar graphs). Results present log2 fold change of OD50 values (for total antibodies) or end-point titer (for anti-VSG antibodies) of infected mice vs naïve sample. For anti-VSG antibodies titers, only significant values when compared with naïve (p≤ 0.05) are presented. *p ≤ 0.05; **p ≤ 0.01, ***p ≤ 0.001 by Student t-test vs naïve values. **(B)** Concentration of TNFα, INFɣ, and IL-10 in blood plasma during infection. DL: detection limit. Data presents mean + SD from 3 individual mice from one representative among three independent experiments. **(C)** IL-10/TNFα and IL-10/INFɣ ratios, computed by dividing the average IL-10 concentration by the average TNF and INFγ concentrations at the same time point.

Antibody-mediated parasite killing, driven by B cells activation, is considered to be the most efficient anti-*T. evansi* immune response (49). To investigate a potential correlation between antibody responses and disease susceptibility, total antibody and anti-VSG specific antibody titers were measured (**Figure 2A**). *T. evansi* Merzouga 93 infected mice first showed a significant induction of total-IgM and IgG2c levels, followed by increased IgG2b and IgG3 titers towards the end of infection. There was no consistent measurable induction of a total IgG1. With respect to anti-trypanosome specific responses, chronic infection was characterized by the induction of an early anti-VSG IgM response, accompanied by the production of anti-VSG IgGs from the second week onwards. This response was heavily biased towards IgG2c.

In parallel to antibody titer measurements, the dynamic balance between systemic secretion of TNFα, INFγ, and IL-10 was monitored (**Figure 2B**). While increased TNFα levels were present from the first week of infection onwards and peaked after 4 weeks, INFγ levels peaked relatively early at 7dpi, following the development of the first parasitemia wave. To counteract the potential damaging effects of the pro-inflammatory cytokine production, infected mice exhibited elevated plasma level of the anti-inflammatory cytokine IL-10. Ratios of IL-10 to TNFα and IL-10 to INFγ show a persistent anti-inflammatory cytokine environment during the chronic stage of infection (**Figure 2C**).

### scRNA-seq analysis reveals a high heterogeneity of macrophage populations during early and late stages of chronic *T. evansi* infections

**Figure 3:**
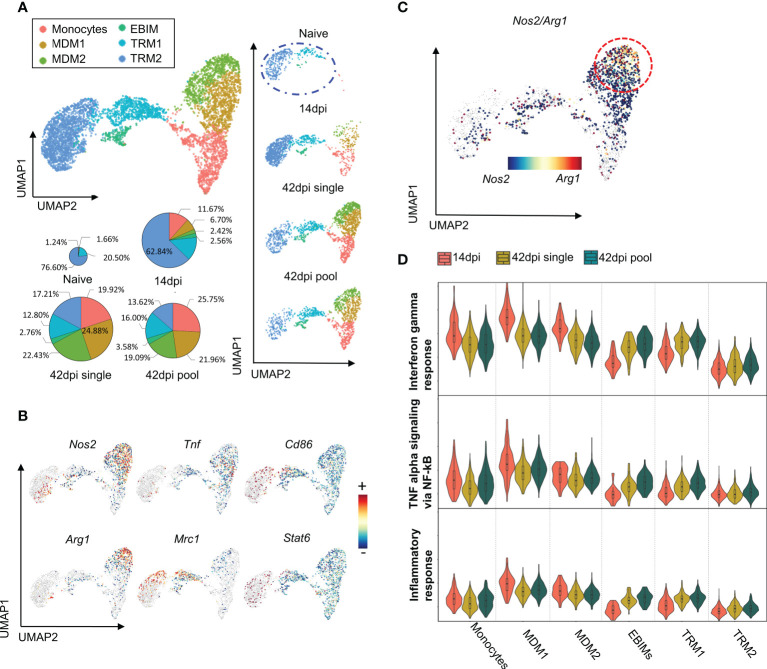
scRNA-seq analysis reveals heterogeneity of Macrophages population during early and late stage of *T. evansi* infection. **(A)** Uniform Manifold Approximation Projection (UMAP) of Macrophages sub-populations and their portion across 3 time points (pie charts). MDM: Monocytes Derived Macrophages, EBIM: Erythroblast Island Macrophages, TRM: Tissue Resident Macrophages. **(B)** Log-normalized expression of classical markers for M1 (upper panel) and M2 (lower panel) macrophages on combined Macrophages from 3 time points. **(C)** Co-expression levels of *Nos2/Arg1* genes on 42dpi single and 42dpi pool datasets derived Macrophages. **(D)** Interferon gamma response score (Top panel), TNF alpha signaling *via* NF-kB score (Middle panel), and Inflammation response score (Bottom panel) of Macrophages sub-populations during infection.

The abovementioned results are consistent with prior studies that have shown a correlation between the chronicity of disease, cytokine environment and macrophage polarization (15, 28, 50). To address this link in chronic *T. evansi* trypanosomosis, a scRNA-seq analysis was performed, capturing the transcriptome profile of macrophages during infection. Experimental samples included splenocytes from naive and *T. evansi* Merzouga 93 infected mice on day14 (acute phase) and day 42 (chronic phase). For the first two time points (non-infected and 14dpi), the sequencing libraries were generated using single individual mice. For the purpose of result validation, two independent libraries were constructed for the 42 dpi timepoint, using either spleen cells from a single mouse (single sample) or cells pooled from three individual mice in a 1:1:1 ratio (pool sample) (**Figures S2A, B**). Merged data from the 3 time points was used to generate a combined dataset, in which a total of 30,321 cells were submitted to unbiased graph-based clustering (Seurat), resulting in 32 distinct clusters (**Figure S2C**). Macrophage populations were manually identified among those clusters, based on canonical markers including *Adgre1, C1qa, C1qb*, and *Fcgr1* (see supplementary gene table for explanatory gene list). Cluster 12 expressing transcription profiles of monocytes (*Ly6c2, Itgam, Ccr2*) and macrophages (*Adgre1, C1qa, C1qb, C1qc*), was included in this analysis as well (**Figure S2D**). Next, these populations were sub-clustered using Seurat, to re-perform dimensionally reduction, yielding 9 discrete sub-clusters (**Figure S3A**). Since clusters 0, 2, 6, and 8 are present in both homeostasis (naïve) and infection derived samples, and have low expression of *Itgam* (CD11b) but high level of genes known to be induced by heme exposure such as *Hmox1* and *Ftl1*, those clusters were annotated as tissue-resident macrophages (TRM1 and TRM2) (**Figures S3A, B**). Clusters 1, 3, 4,5 are hardly present in the naïve dataset, but are highly induced upon infection, with two clusters (4 and 5) annotated as monocytes expressing high levels of monocyte-specific genes like *Ly6c2*, *Plac8*, and *Itgam* (CD11b) and low levels of macrophage-specific genes like *Adgre1* (F4/80) or *C1qc*. The other two clusters (1 and 3) were annotated as monocytes-derived-macrophages (MDM) 1 and 2, respectively, since they express high levels of macrophages markers, while maintaining constant level of monocyte markers. Finally, a small cell population (cluster 7) was identified as Erythroblast Island macrophages (EBIM), since these cells express signature genes such as *Hbb-bs, Alas2*, and *Gypa* (Ter119**)** (51). Based on these annotations, infection-induced alterations in the size of each macrophage subpopulations was calculated in absolute cell numbers, showing their accumulation upon infection. During homeostasis, monocytes and monocyte-derived macrophages only account for 2.9% of total splenic macrophages, but these sub-populations rose exponentially during infection, accounting for 20.79% at 14dpi, and 66.8%/67.23% (pool/single) at the 42dpi chronic stage of infection (**Figure 3A**). The tissue resident macrophage subpopulation predominated in the naïve and 14 dpi samples, declining in size at the chronic 42 dpi time point. While EBIMs were virtually absent from the naïve population, their frequency increased to around 2.44% at 14dpi and 2.76%/3.58% 42dpi.

Next, an attempt was made to identify the subpopulation of M1 and M2 macrophages. Surprisingly, applying the most commonly used markers expression *Nos2, Tnf, Cd86* for M1 and *Arg1, Mrc1*, *Stat6* for M2, did not result in the identification of a distinct cluster (**Figure 3B**). In contrast, the two MDM1 and MDM2 subpopulations simultaneously express both the M1-specific gene marker *Nos2*, encoding iNOS, and the M2-specific gene marker *Agr1*, encoding Arginase, despite the fact that these enzymes have been described as M1 and M2 exclusion indicators, respectively (52). Additionally, the TRM1 subpopulation and part of the TRM2 simultaneously express genes coding for CD86, the co-stimulatory molecule on M1 macrophages and CD206 (*Mrc1*), a common marker to identify the M2 phenotype in both naïve and inflammatory conditions, even though cells should not be activated/polarized during homeostasis (**Figure 3B**, **Figure S3C**). An UMAP showing signature score as the mean expression of comprehensive classic gene markers for M1/M2 macrophages acquired from the literature showed mixed phenotype of M1 and M2 subpopulations in our dataset (supplementary gene table, **Figure S3D**). Even at the chronic stage of infection (42dpi), which is expected to have a M2 macrophages bias, cells from monocyte-derived macrophage subpopulation are *Nos2/Arg1* double positive, indicating a mix phenotype that cannot be categorized along the M1/M2 paradigm lines (**Figure 3C**).

To characterize the macrophage sub-types in detail, result from DEG analysis were used to perform a gene set enrichment analysis for each clusters. MDM1 and MDM2 subpopulations express genes enriched for INFɣ responses, TNF signaling *via* NF-κB, and inflammatory responses, suggesting the immune stimulating function of these cells during infection (**Figure 3D**). While MDM1s have the highest signature score for pro-inflammatory processes, exhibiting an increased *Nos2/Arg1* double positive cell characteristic at 42dpi, this signature is significantly reduced when compared to 14dpi (**Figure 3D**).

### TRMs and EBIM contribute to regulation of infection-induced splenic extramedullary erythropoiesis

**Figure 4:**
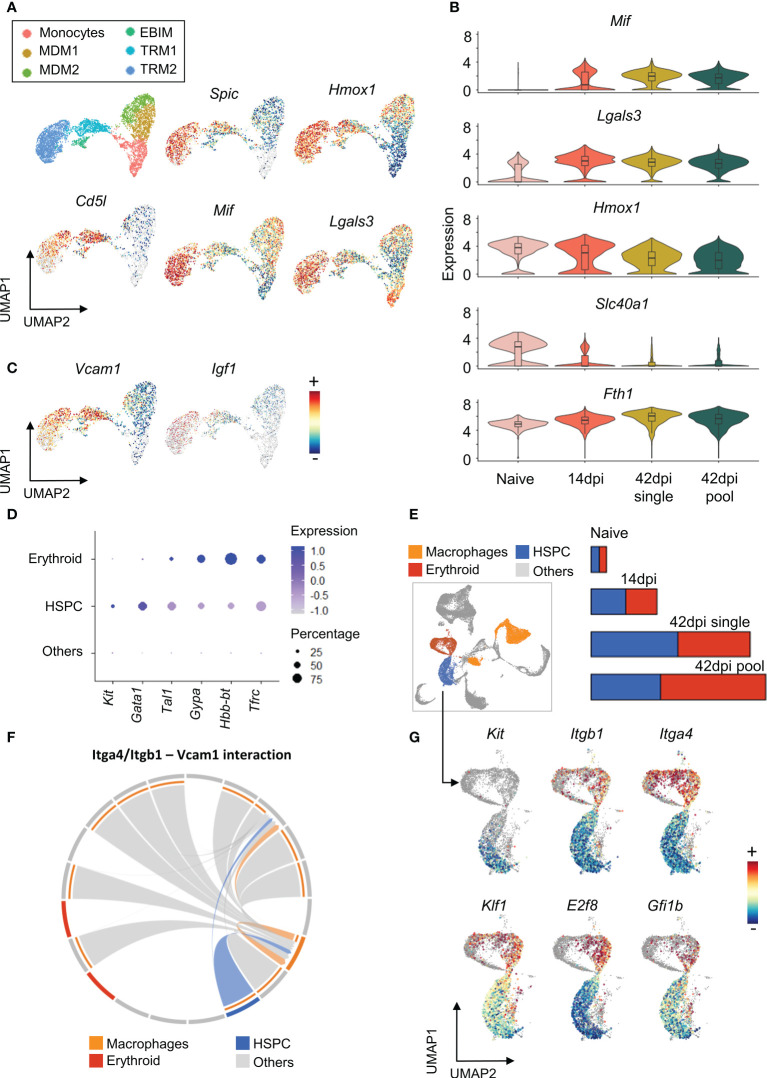
Tissue resident macrophages show multi potential roles during chronic *T. evansi* infection. **(A)** UMAP showing macrophages sub-population (first panel) and log-normalized expression of genes expressed by Tissue resident macrophages **(B)** Changes in expression of genes related to iron retention during infection. **(C)** UMAP showing expression of *Vcamp1* and *Igf1* genes. **(D)** Average expression of gene markers used for HSPCs and Erythroid annotation. **(E)** UMAP projection of Macrophages, HSPCs, and Erythroid populations on combined total spleen cells dataset and their cell number alteration during infection. **(F)** Chord diagram of the inferred Itga4/Itgb1 – Vcam1 interaction among cell populations. **(G)** Log-normalized expression of genes involved in Hematopoiesis and Erythropoiesis on HSPCs and Erythroid populations.

As TRMs dominate the total macrophages population in both non-infected and 14 dpi spleen samples (**Figure 3A**), and express lower level of inflammation related pathways (**Figure 3D**), the function of these subpopulations was investigated in more details *via* their transcriptional profiles. Differential expression analysis reveals that both TRM1 and TRM2 clusters exhibit high expression level of signature genes for the erythrophagocytic process, including the heme-inducible genes *Spic* and *Hmox1* (**Figure 4A**). Moreover, the expression of the gene coding for the macrophage apoptosis inhibitor i.e. *Cd5l*, was found to be elevated in TRM1 and part of the TRM2 cluster. During infection, the TRM2 cluster strongly expresses the M1-associated Macrophage migration Inhibitory Factor gene *Mif*, and Galectin-3 gene *Lgals3*, that are actively involved in trypanosomosis associated anemia (53). TRM2s also maintain the high expression of *Hmox1* and *Fth1*, coding for Ferritin (heavy chain 1) involved in iron storage (**Figure 4B**). These observations coincide with an infection-induced reduction in expression of the *Slc40a1* gene (**Figure 4B**), encoding the iron-regulated transporter Ferroportin. Taken together, results indicate that TRM2s are involved in induction of infection-associated anemia with a high erythrophagocytic phenotype.

Of note is that the TRMs and EBIM subpopulations are characterized by their high expression of *Vcam1* (**Figure 4C**), encoding the vascular cell adhesion molecule (VCAM-1) used by red pulp resident macrophages to retain hematopoietic stem and progenitor cells (HSPCs) in the spleen. Here, VCAM-1 interacts with HSPCs *via* Integrin α4β1, a heterodimer integrin encoded by the *Itga4* and *Itgb1* genes. As a result, red pulp resident macrophages act as “usher cells” to anchor HSPCs in the spleen (54). This phenomenon is observed under inflammation condition as an “emergency hematopoiesis” mechanism. Interestingly, our combined spleen cells scRNA-seq dataset demonstrates HSPCs accumulation during infection, with the population being characterized by increased expression of the hematopoietic stem cell and progenitor cell transcription factor genes *Kit*, *Gata1*, and *Tal1* (55)(**Figures 4D, E**). Intercellular communication analysis using the Cellchat package, which examines cell-cell contact based on ligands and receptors expression, also reveals a potential interaction between *Itga4/Itgb1* on HSPCs and *Vcam1* on resident macrophages (**Figures 4E, F**). As infection-induced HSPCs also exhibit elevated expression level of genes involved in erythroid differentiation, such as *Klf1*, *E2f8*, and *Gfi1b* (56–58)(**Figure 4G**), they are most likely involved in the process of infection-induced extramedullary erythropoiesis in the spleen. This finding corresponds to the size increase of the erythroid population during infection, annotated by the specific gene markers *Gypa*, *Hbb-bt*, and *Tfrc* (**Figures 4D, E**). Finally, it is worth mentioning that EBIM and TRM2 are increased for the gene encoding Insulin-like growth factor 1 (*Igf1*) (**Figure 4C**), which has been reported to be produced by macrophages to promote erythroid cells proliferation (59). Hence, combined evidences indicates that *T. evansi*-induced TRMs and EBIMs all play a central role in infection-induced extramedullary erythropoiesis.

### Truncated extramedullary erythropoiesis at the level of orthochromatic erythrocyte contributes to the induction of severe anemia and a chronic wasting syndrome

**Figure 5:**
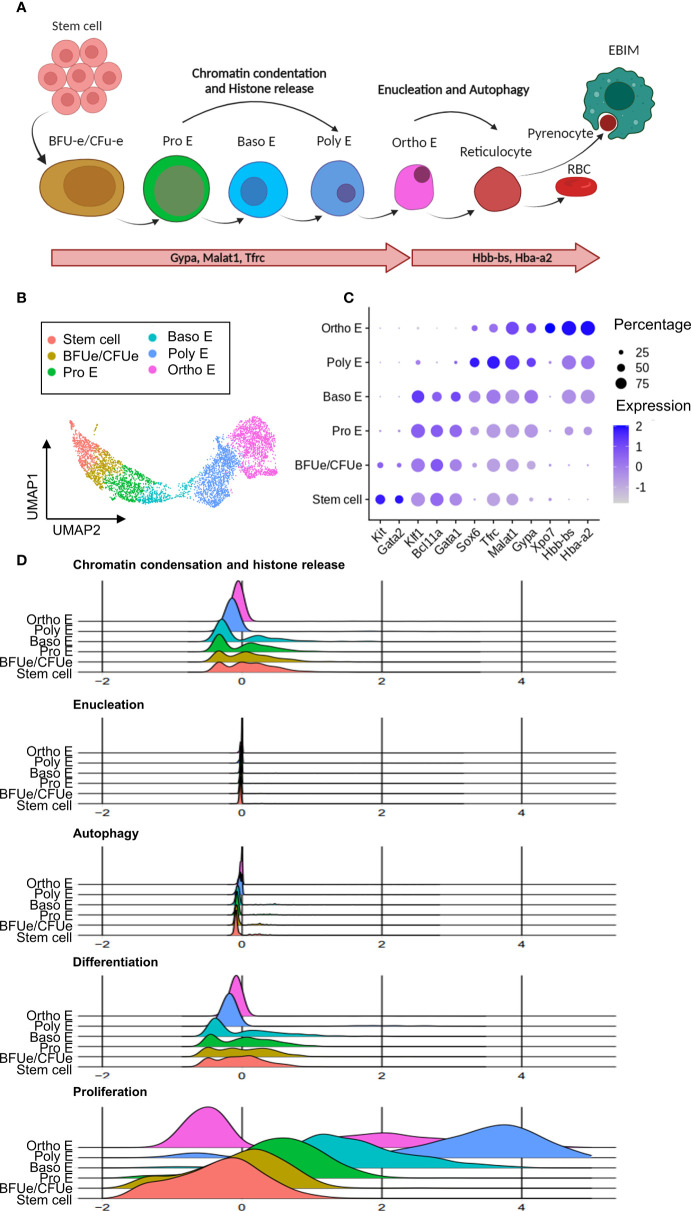
Chronic *T. evansi* infection causes truncation of compensatory extramedullary erythropoiesis. **(A)** Erythropoiesis differentiation process. **(B)** UMAP showing erythrocytes lineage populations in spleen during infection. **(C)** Average expression of gene markers used for Erythrocytes sup-populations annotation. **(D)** Signature score of pathways related to erythropoiesis.

According to the scRNA-seq analysis outlined above, tissue resident macrophages are involved in both infection-induced anemia and infection-associated extramedullary erythropoiesis. To fully understand the mechanisms underlying this pathological situation, the HSPCs and erythroid cells were re-subjected to a dimensional reduction and clustering analysis. This resulted in 7 distinct clusters, annotated according to known erythropoiesis differentiation stages. Details of these maturation stages (**Figure 5A**) show stem cells that have a high expression of the growth factor receptor, *Kit*, and the stem cell maintenance transcription factor *Gata2.* Next, the extramedullary erythropoiesis process is triggered by the presence of early and late erythroid precursor cells (BFU-e and CFU-e). These cells have increased expression of the transcription factors *Klf1*, *Bcl11a*, and *Gata1*, which are required for early steps of erythroid differentiation. The subsequent stages of erythropoiesis, which include pro-erythroblast (Pro E), basophilic erythroblast (Baso E), polychromatophilic erythroblast (Poly E), and orthochomatic erythroblast (Ortho E), are distinguished by gradually increased expression of erythroid maturation genes such as *Sox6, Tfrc*, *Malat1*, and *Gypa*. The latter is also identified by the expression of the erythroid highly specialized *Xpo7* gene, hemoglobin coding genes *Hbb-bs* and *Hba-a2*. The ‘Ortho E’ fraction is the last step of erythropoiesis that can be captured by scRNA-seq in our dataset, as it represents the last pre-reticulocyte stage before the enucleation takes place (**Figures 5B, C**).

During terminal differentiation to reticulocytes, erythroblasts undergo multiple processes by which cells prepare for the process of enucleation and removal of other organelles, to obtain fully mature red blood cell features (**Figure 5A**). As demonstrated in **Figure 5D**, genes that related to these processes (supplementary gene table) were used to calculate “Chromatin condensation and histone release”, “Enucleation”, and “Autophagy” signature scores during chronic stage of *T. evansi* Merzouga 93 infection. Surprisingly, Pro E, Baso E, and Poly E, the three differentiation stages during which cells are expected to express a high level of genes associated to chromatin condensation and histone release, have a low to average signature score for this process, while none of the clusters had an enriched score for autophagy and enucleation (**Figure 5D**, first 3 panels). Similarly, a signature score was calculated using genes that were reported to have positive regulatory function on terminal erythropoiesis differentiation (**Figure 5D**, 4^th^ panel). While those regulators have an enriched score in part of the stem cell, BFU-e/CFU-e, Pro E, and Baso E subpopulations, they do not appear to be expressed in the later stages of erythropoiesis, indicating that chronic stage of *T. evansi* infection is characterized by a truncated reticulocyte generation, as cells are unable to pass the orthochromatic E stage. Furthermore, instead of being positively stimulated to differentiate towards the Ortho E stage, cells in the Pro E, Baso E, and Poly E subpopulations show elevated proliferation scores (**Figure 5D**, last panel). As such, these events are shifting the balance of erythropoiesis toward early-stage proliferation rather than final-stage differentiation, blocking reticulocyte release and mature RBCs production.

**Figure 6:**
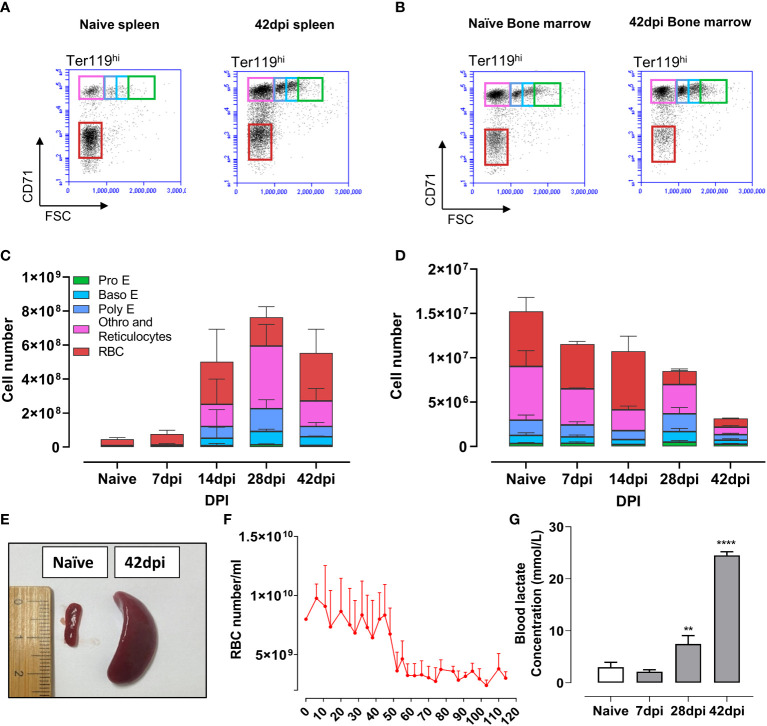
Truncated extramedullary erythropoiesis failed to compensate for the loss of erythrocytes lineages pool in bone marrow, resulting in anemia followed by lactic acidosis. **(A, B)** Erythrocyte lineage cells in spleen and bone marrow by flow cytometry analysis at naïve and 42dpi. Results are a representative data of a triplicate repeat experiment with 3 mice. **(C, D)** Absolute number of erythrocyte linage in spleen and bone marrow during infection obtain by flow cytometry analysis. **(E)** Splenomegaly observed during infection. **(F)** Number of red blood cell during infection. Data presents mean + SD from 5 individual mice. **(G)** Blood lactate concentration of mice during infection. Data presents mean + SD from 3 individual mice. **p ≤ 0.01; ****p ≤ 0.0001 by Student t-test vs naive values.

To corroborate the observation of the infection-induced extramedullary erythropoiesis arrest at the cellular level, flow cytometry was used to quantify the number of erythroid cells in the spleen and bone marrow of infected mice (**Figures 6A–D**). During infection, extramedullary erythropoiesis was observed in the spleen, characterized by an increase in the number of cells at all differentiation stages. However, these numbers decreased in the bone marrow as the infection progressed. Baso E, Poly E, and Ortho E, three populations having a high proliferation score in the scRNA-seq analysis, make up the majority of cells that expanded in the spleen after infection, demonstrating an imbalance in favor of proliferation of these cells over differentiation into mature RBCs. As a consequence, accumulation of truncated erythropoiesis, the primary cause of splenomegaly (**Figure 6E**), was unable to compensate for the loss of erythrocytes pool in bone marrow, resulting in persistent anemia during infection (**Figure 6F**). Chronic anemia often results in hypoxia due to the inability to oxygenate all organs and tissues. The latter can be a major cause of lactic acidosis as a consequence of anaerobic glycolysis (60). As such, the blood lactate concentration of infected mice was determined. Remarkably, during the chronic stage of infection, lactate concentration increased exponentially, reaching 8 times its normal level (**Figure 6C**). These results indicate that during chronic *T. evansi* Merzouga 93 infections, extreme systemic lactic acidosis is coinciding with the catabolic muscle disorder (**Figure 1C**) that hallmarks the final lethal stage of the infection.

## Discussion

In domestic livestock animals, *T. evansi* infections are is known to cause severe anemia accompanied by a catabolic muscle wasting syndrome, despite parasitemia levels being well-controlled during the chronic stage of infection. In order to identify a suitable experimental study model that would reflect diseases outcomes observed in *T. evansi* AT, 10 infection mouse models were set up using non-cloned isolates from various geographic locations and hosts. Obtained results allowed to classify these isolates in four distinct groups with variable degrees of virulence. Rapidly progressing hyper-virulent and virulent infections both resulted in mice being unable to control high parasitemia, quickly reaching sever morbidity. A third intermediate phenotype group exhibited slightly prolonged infection of up to 5 weeks, but still suffered from excessive end-stage parasitemia. The fourth group, represented by *T. evansi* Merzouga 93, showed a long lasting well-controlled parasitemia profile. This infection induced a chronic wasting syndrome, characterized by profound anemia and a severe reduction in muscle mass. In the current study, these two phenomena were linked for the first time by the finding of extreme systemic lactic acidosis during chronic infection, making the Merzouga 93 model suitable to investigate the mechanisms of *T. evansi* associated lethal pathology.

*T. evansi* Merzouga 93 infected mice are able to maintain low numbers of parasites in the blood for well over 100 days, in the presence of anti-VSG antibodies. Previously we have shown that IgMs are crucial for *T. evansi* parasitemia control (49) and that AID^-/-^ mice, which are unable to produce IgGs, are maintaining low blood parasite numbers during chronic infection (10). Besides antibodies, other immune components such as macrophages can directly be involved in trypanosome parasite control, through phagocytosis involving the CR3 receptor (61) or CRIg/VSIG4 (62). Macrophage activation also induces an inflammatory cytokine environment with high levels of TNF and NO, which impair both *T. brucei* and *T. congolense* parasite survival (22–24, 63). However, by using INFγ^-/-^, TNF^-/-^, and TNFR^-/-^ mice, it has been shown that the latter does not occur in case of *T. evansi* (49). It is possible that other parasite density control mechanisms are involved here. While quorum sensing has so far only been described in detail to occur in *T. brucei* (64, 65), it is worth speculating that at least in some of the more chronic *T. evansi* infections, a similar mechanism could ensure prolonged host survival, even under conditions where B cell immune suppression takes place. In all trypanosomosis models published so far, the infection-associated excessive production of pro-inflammatory cytokines was shown to be counterbalanced by anti-inflammatory IL-10 response. This, in turn, prevents the death of the host by uncontrolled hyper-inflammation (28, 66). In line with this, our present study shows that maintaining high systemic IL-10 level coincides with prolonged survival time in chronically *T. evansi* infected mice.

Previous AT studies have frequently correlated the disease-induced cytokine environment to alterations in macrophage polarization, i.e. the occurrence of classically activated so-called M1 and alternative activated so-call M2 macrophages (16, 18). However, this bipolar paradigm, which was initially established using *in vitro* stimulated macrophages (67, 68), has often been based on bulk RNA sequencing representing ‘whole-organ’ cytokine expression levels. In the setting of trypanosomosis, the ratio between M1/M2 macrophages has been examined in the past using readouts such as levels of nitrite for nitric oxide synthase activity and arginase activity, respectively (27, 69). Alternatively, the expression of a limited number of genes, thought to be exclusive indicators of M1 or M2 macrophages, was used to assess organ level gene expression using reverse transcriptase quantitative PCR (RTqPCR) (14, 15, 28, 70, 71). To date, it has however become possible to assess the heterogeneity of macrophages in the tissue environment at a single-cell resolution, using the scRNA-seq approach. As a result, emerging evidence suggests that macrophage programming *in vivo* is multimodal, tissue and context- specific, and should no longer be restricted to a strict bipolar interpretation (29, 72). In line with this, our scRNA-seq dataset indicates that macrophage population composition in the spleen is complex, during homeostasis as well as during progressing *T. evansi* infections, but cannot be simply subdivided according to the M1/M2 dichotomy model. While our scRNA-seq data shows existence of two subpopulations of monocyte-derived macrophages (MDM1 and MDM2), there was no apparent segregation between M1 and M2 macrophages, neither using conventional genes nor a combined list of genes resulting from earlier research based on flow cytometry. Both infection-induced MDM1 and MDM2 populations have high signature scores for pro-inflammatory pathways such as an INFγ response, TNFα signaling *via* NF-kB, and general inflammatory responses. Interestingly, cells from these subpopulations co-express simultaneously the *Nos2* and *Arg1* genes, which have been extensively used as exclusion markers to distinguish M1 from M2 macrophages (52). The existence of macrophages co-expressing *Nos2* and *Arg1* genes, exerting immunosuppressive phenotype, has also been described in other parasite infections such as *Trypanosoma cruzi* or *Giardia lamblia*, as well as during bacterial *Mycobacterium tuberculosis* infection (73–75). Hence, although defining a new macrophage classification is not the primary goal of the work presented here, we recommend reconsidering the concept of attributing the bipolar macrophage paradigm as the cause of cytokine balance regulation, as this appears to be oversimplified at least in the context of trypanosomosis.

To date, it is well documented that mammalian tissues have their own TRMs, which are essential for maintaining homeostasis as well as exerting specific immunological functions (76, 77). Using *T. brucei* and *T. congolense* infection models, it has been reported that Kupffer cells, the TRMs of the liver, play an essential role in clearance of trypanosomes by CRIg-mediated parasites phagocytosis (62, 78). The current study found that during *T. evansi* trypanosomosis, TRMs of the spleen have an enhanced erythrophagocytosis signature, marked by high expression of genes encoding macrophage migration inhibitory factor (MIF) and Galectin-3. These molecules contribute to infection-induced anemia, as Gal-3 induce erythrophagocytosis, while MIF promotes the production of pro-inflammatory cytokines, including TNF (31, 32). Additionally, parasite infection enhanced the iron retention phenotype of splenic macrophages, as demonstrated here by the downregulation of *Slc40a1* gene, encoding the cell surface iron exporter ferropotin, and the upregulation of *Fth1 gene, coding for* the major intracellular iron storage protein ferritin. In the context of infection, iron retention is a process that is often believed to help the host in inhibiting pathogen growth, by restricting their supply of nutrients. However, in the context of AT it is doubtful that this strategy would be efficient, as trypanosomes have multiple receptor-dependent and independent mechanism to scavenge iron (79). In contrast, AT-induced iron retention could be considered a very dangerous double-edged sword, because it inhibits erythropoiesis in the bone marrow, by lack of iron availability (80). Interestingly, while this occurs, infection-induced inflammation can actively promote compensatory extramedullary erythropoiesis (81). In the current study, the occurrence of the latter is supported by scRNA-seq analysis. During infection splenic TRMs highly express *Hmox1* gene, coding for Heme oxygenase enzyme. As the breakdown of hemoglobin after RBC clearance releases heme, the expression of *Hmox1* is increased, as well as the expression of the heme-dependent transcription factor SpiC. Hence, as *T. evansi* Merzouga 93 infection suppresses bone marrow erythropoiesis, the spleen gene regulation pattern signals for the activation of extramedullary erythropoiesis, in order to maintain erythroid homeostasis (82). In this context, our data show that TRMs along with EBIMs highly express *Vcam1*, an adhesion molecule retaining hematopoietic stem and progenitor cells, suggesting their involvement in extramedullary erythropoiesis. As the increase in EBIMs coincides with the expansion of HSPCs and the overall erythroid population in the spleen, HSPCs were further sub-clustered into distinct sub-populations using the scRNA-seq data set, in order to identify and characterize different cells undergoing erythrocyte differentiation. In our dataset, the orthochromatic erythrocyte stage is the last stage of differentiation that can be captured by transcriptomic analysis, as cells prepare for terminal differentiation into mature RBCs by removal of the nucleus *via* enucleation (38). These cells also remove their organelles through autophagy (83). Surprisingly, during the chronic stage of the *T. evansi* Merzouga 93 infection, the orthochromatic erythrocytes show no elevated signature scores for genes related to enucleation or autophagy. In addition, early-stage erythroid cells did not exhibit signs of chromatin condensation and histone release, which are required for the final-stage enucleation event (84). In contrast, erythroid progenitors show a transcriptomic bias towards proliferation. Hence, the current data demonstrates that abrogation of terminal differentiation of orthochromatic erythroblasts into enucleated reticulocytes, prevents the successful replenishment of mature RBCs. Subsequently, failure of generating RBCs through the spleen that could compensate for the loss of bone marrow derived-RBCs, results in persistent *T. evansi* Merzouga 93 induced anemia. It is worth noting that in AT, the induction of anemia is independent of the concentration of erythropoietin (EPO) (85–87).

Combined, our findings are particularly important in the context of trypanosomosis, because it allows to speculate on the true nature of the lethal infection-associated pathology of chronic *T. evansi* Merzouga 93 and AT, by linking the induction of anemia to the development of a catabolic muscle dystrophy wasting syndrome. Indeed, it is generally known that anemia impairs oxygen transport capacity which results in tissue hypoxia (88). In turn, hypoxia triggers the induction of EPO in kidney, which drives the expansion and differentiation of erythroid progenitors (89). In case of insufficient bone marrow erythropoiesis, hypoxia triggers extramedullary erythropoiesis to rapidly generate additional RBCs, compensating and maintaining erythrocytes homeostasis (90). However, *T. evansi* Merzouga 93 infection disrupts these homeostatic processes by the combination of (i) excessive erythrophagocytosis, (ii) inflammation-induced inhibition of bone marrow erythropoiesis, and (iii) arrested extramedullary erythropoiesis, due to truncation of orthochromatic erythrocytes differentiation. As chronic anemia can result in hypoxia due to the inability to oxygenate all organs and tissues, we measured the blood lactate concentration, the end product of anaerobic glycolysis. Results show that during the chronic stage of *T. evansi* Merzouga 93 infection, lactate concentration exponentially increased, reaching 8 times its normal level. Interestingly lactic acidosis has also been directly to iron deficiency (91) as well as to the metabolic activity of highly proliferating erythroid progenitors and immune cells (92–94). Important is that lactic acidosis itself has been linked to catabolic muscle degradation through several mechanisms, including (i) inhibition of the glycolytic flux in skeletal muscles, by blocking skeletal hexokinase and phosphofructokinase, leading to inhibition of the carbohydrate metabolism itself (95, 96), (ii) pain-induced reduced locomotor activity (97, 98), resulting in reduced food intake, (iii) the breakdown of amino acids through the glutamine/glutamate/α-ketoglutarate and aspartate/oxaloacetate shuttles that feeds proteins into the Krebs-cycle allowing NADH/ATP generation under starvation conditions, and (iv) the association with chronic kidney disease and kidney failure (99, 100). Combined, these factors aggravate each other as they drive a perpetuated cycle that can quickly deteriorate, and could explain the ‘multi-organ failure’ that is often quoted as the cause of death in livestock trypanosomosis (16, 32). Interestingly, while a single publication on a human trypanosomosis case did report the occurrence of lactic acidosis (101), to our knowledge there are no studies documenting blood acidosis measurements in the context of AT. Hence, the results presented in this work are unique, in that they show an extreme increase of blood lactate concentration toward the end of chronic *T. evansi* infection. As such, we suggest that monitoring blood lactate concentration in infected animals could serve as an indicator for severity of the disease. In the context of trypanosomosis, it is important to flag that the parasite itself can also produce L-lactate, identical to the lactate isoform produced by mammals (102, 103). However, our study shows that high level of blood lactate were present in infected mice, despite well-controlled parasitemia at the end of infection. This suggests that the host’s derived factors dominate this pathology. Interestingly, elevated concentration of lactate have been shown to be harmless to trypanosomes, and hence should not be considered a factor that contributes to the maintenance of low parasitemia levels during the chronic stage infection (104).

In conclusion, our study used multiple non-cloned field isolates of *T. evansi* to evaluate the immunopathology of chronic animal trypanosomosis. First, our results show that chronic *T. evansi* infection is characterized by a prolonged phase of well-controlled parasitemia, even under immune conditions where the host B cell compartment is severely affected (10). Secondly, our data confirms that increased anti-inflammatory IL-10 production coincides with prolonged survival of trypanosome infected mice. However, we suggest reconsidering the link between trypanosomosis-induced cytokine balances and the M1/M2 macrophage dichotomy. The latter seem to be over-simplified, without consideration of tissue resident macrophage function. Finally, our study links AT-induced catabolic muscle wasting and extreme lactic acidosis, with anemia that involves the abrogation of extramedullary erythropoiesis at the level of the orthochromatic erythrocyte differentiation.

## Data availability statement

scRNA sequencing (scRNA-seq) datasets used in this study are publicly available in the BioStudies EMBL-EBI database (www.ebi.ac.uk/biostudies). The datasets can be accessed under the accession numbers E-MTAB-10174, which originated from https://doi.org/10.1371/journal.ppat.1010026, and E-MTAB-12109.

## Ethics statement

The animal study was reviewed and approved by The Ghent University Global Campus Institutional Animal Care (GUGC IACUC).

## Author contributions

Conceptualization, MR, SM, and HN, Investigation, HN, MR, and SM. Data analysis and interpretation, HN, MR, and SM. Writing, HN, SM, and MR. Critical revision, MR and SM. Visualization, HN. Funding acquisition, SM and MR. All authors contributed to the article and approved the submitted version.

## Funding

This work was supported by Ghent University Global Campus core funding, UGent BOF grant number BOF.STG.2018.0009.01/01N01518 and FWO grant number G013518N.

## Acknowledgments

We thank Dr. Zeng Li for her technical support related to Rotat 1.2 gene detecting using PCR, Dr. Daliya Kancheva for discussion on Macrophages scRNA-seq data annotation. We also would like to thank Bolortsetseg Baatar and Boyoon Choi for their assistance in samples preparation.

## Conflict of interest

The authors declare that the research was conducted in the absence of any commercial of financial relationships that could be construed as a potential conflict of interest.

## Publisher’s note

All claims expressed in this article are solely those of the authors and do not necessarily represent those of their affiliated organizations, or those of the publisher, the editors and the reviewers. Any product that may be evaluated in this article, or claim that may be made by its manufacturer, is not guaranteed or endorsed by the publisher.

## References

[1] Zweygarth E Kaminsky R. *In vitro* differentiation between *Trypanosoma brucei brucei* and *T.b. evansi* . Trop Med Parasitol (1989) 40:115–11. 2772515

[2] Vanhollebeke B Truc P Poelvoorde P Pays A Joshi PP Katti R. Human *Trypanosoma evansi* infection linked to a lack of apolipoprotein l-I. N Engl J Med (2006) 355:2752–6. doi: 10.1056/NEJMoa063265 17192540

[3] Truc P Buscher P Cuny G Gonzatti MI Jannin J Joshi P. Atypical human infections by animal trypanosomes. PloS Negl Trop Dis (2013) 7:e2256. doi: 10.1371/journal.pntd.0002256 24069464 PMC3772015

[4] Van Vinh Chau N Buu Chau L Desquesnes M Herder S Phu Huong Lan N Campbell JI. A clinical and epidemiological investigation of the first reported human infection with the zoonotic parasite *Trypanosoma evansi* in southeast Asia. Clin Infect Dis (2016) 62:1002–8. doi: 10.1093/cid/ciw052 PMC4803109 26908809

[5] Aregawi WG Agga GE Abdi RD Buscher P. Systematic review and meta-analysis on the global distribution, host range, and prevalence of *Trypanosoma evansi* . Parasit Vectors (2019) 12:67. doi: 10.1186/s13071-019-3311-4 30704516 PMC6357473

[6] Desquesnes M Dargantes A Lai DH Lun ZR Holzmuller P Jittapalapong S. *Trypanosoma evansi* and surra: a review and perspectives on transmission, epidemiology and control, impact, and zoonotic aspects. BioMed Res Int (2013) 2013:321237. doi: 10.1155/2013/321237 24151595 PMC3789323

[7] Gutierrez C Tamarit A Gonzalez-Martin M Tejedor-Junco MT. Control and eventual eradication of *Trypanosoma evansi* infection in dromedary camels after an episodic outbreak in mainland Spain: an example in a non-endemic area. Vet Parasitol (2014) 204:153–7. doi: 10.1016/j.vetpar.2014.05.004 24933467

[8] Desquesnes M Holzmuller P Lai DH Dargantes A Lun ZR Jittaplapong S. *Trypanosoma evansi* and surra: a review and perspectives on origin, history, distribution, taxonomy, morphology, hosts, and pathogenic effects. BioMed Res Int (2013) 2013:194176. doi: 10.1155/2013/194176 24024184 PMC3760267

[9] Bangs JD. Evolution of antigenic variation in African trypanosomes: Variant surface glycoprotein expression, structure, and function. Bioessays (2018) 40:e1800181. doi: 10.1002/bies.201800181 30370931 PMC6441954

[10] Nguyen HTT Guevarra RB Magez S Radwanska M. Single-cell transcriptome profiling and the use of AID deficient mice reveal that b cell activation combined with antibody class switch recombination and somatic hypermutation do not benefit the control of experimental trypanosomosis. PloS Pathog (2021) 17:e1010026. doi: 10.1371/journal.ppat.1010026 34762705 PMC8610246

[11] Verdillo JC Lazaro JV Abes NS Mingala CN. Comparative virulence of three *Trypanosoma evansi* isolates from water buffaloes in the Philippines. Exp Parasitol (2012) 130:130–4. doi: 10.1016/j.exppara.2011.11.006 22154978

[12] Kamidi CM Auma J Mireji PO Ndungu K Bateta R Kurgat R. Differential virulence of camel *Trypanosoma evansi* isolates in mice. Parasitology (2018) 145:1235–42. doi: 10.1017/S0031182017002359 PMC6057853 29362015

[13] Onyilagha C Uzonna JE. Host immune responses and immune evasion strategies in African trypanosomiasis. Front Immunol (2019) 10:2738. doi: 10.3389/fimmu.2019.02738 31824512 PMC6883386

[14] Baetselier PD Namangala B Noël W Brys L Pays E Beschin A. Alternative versus classical macrophage activation during experimental African trypanosomosis. Int J Parasitol (2001) 31:575–87. doi: 10.1016/S0020-7519(01)00170-9 11334945

[15] Noël W Hassanzadeh G Raes G Namangala B Daems I Brys L. Infection stage-dependent modulation of macrophage activation in *Trypanosoma congolense*-resistant and -susceptible mice. Infect Immun (2002) 70:6180–7. doi: 10.1128/IAI.70.11.6180-6187.2002 PMC130440 12379696

[16] Stijlemans B Guilliams M Raes G Beschin A Magez S De Baetselier P. African Trypanosomosis: from immune escape and immunopathology to immune intervention. Vet Parasitol (2007) 148:3–13. doi: 10.1016/j.vetpar.2007.05.005 17560035

[17] Kuriakose SM Singh R Uzonna JE. Host intracellular signaling events and pro-inflammatory cytokine production in African trypanosomiasis. Front Immunol (2016) 7:181. doi: 10.3389/fimmu.2016.00181 27242788 PMC4872169

[18] Stijlemans B De Baetselier P Magez S Van Ginderachter JA De Trez C. African Trypanosomiasis-associated anemia: The contribution of the interplay between parasites and the mononuclear phagocyte system. Front Immunol (2018) 9:218. doi: 10.3389/fimmu.2018.00218 29497418 PMC5818406

[19] Hertz CJ Filutowicz H Mansfield JM. Resistance to the African trypanosomes is IFN-gamma dependent. J Immunol (1998) 161(12):6775–83. 9862708

[20] Magez S Radwanska M Beschin A Sekikawa K De Baetselier P. Tumor necrosis factor alpha is a key mediator in the regulation of experimental *Trypanosoma brucei* infections. Infect Immun (1999) 67:3128–32. doi: 10.1128/IAI.67.6.3128-3132.1999 PMC96631 10338530

[21] Musaya J Matovu E Nyirenda M Chisi J. Role of cytokines in *Trypanosoma brucei*-induced anaemia: A review of the literature. Malawi Med J (2015) 27:45–50. doi: 10.4314/mmj.v27i2.3 26405511 PMC4562079

[22] Duleu S Vincendeau P Courtois P Semballa S Lagroye I Daulouède S. Mouse strain susceptibility to trypanosome infection: an arginase-dependent effect. J Immunol (2004) 172:6298–303. doi: 10.4049/jimmunol.172.10.6298 15128819

[23] Magez S Radwanska M Drennan M Fick L Baral TN Brombacher F. Interferon-gamma and nitric oxide in combination with antibodies are key protective host immune factors during *trypanosoma congolense* Tc13 infections. J Infect Dis (2006) 193:1575–83. doi: 10.1086/503808 16652287

[24] Lu W Wei G Pan W Tabel H. Trypanosoma congolense infections: Induced nitric oxide inhibits parasite growth *In vivo* . J Parasitol Res (2011) 2011:316067. doi: 10.1155/2011/316067 21584233 PMC3092548

[25] Alfituri OA Quintana JF Macleod A Garside P Benson RA Brewer JM. To the skin and beyond: The immune response to African trypanosomes as they enter and exit the vertebrate host. Front Immunol (2020) 11:1250. doi: 10.3389/fimmu.2020.01250 32595652 PMC7304505

[26] Guilliams M Movahedi K Bosschaerts T Vandendriessche T Chuah MK Hérin M. IL-10 dampens TNF/inducible nitric oxide synthase-producing dendritic cell-mediated pathogenicity during parasitic infection. J Immunol (2009) 182:1107–18. doi: 10.4049/jimmunol.182.2.1107 19124754

[27] De Muylder G Daulouède S Lecordier L Uzureau P Morias Y Van Den Abbeele J. A *Trypanosoma brucei* kinesin heavy chain promotes parasite growth by triggering host arginase activity. PloS Pathog (2013) 9:e1003731. doi: 10.1371/journal.ppat.1003731 24204274 PMC3814429

[28] Namangala B Noël W De Baetselier P Brys L Beschin A. Relative contribution of interferon-gamma and interleukin-10 to resistance to murine African trypanosomosis. J Infect Dis (2001) 183:1794–800. doi: 10.1086/320731 11372033

[29] Martinez FO Gordon S. The M1 and M2 paradigm of macrophage activation: time for reassessment. F1000Prime Rep (2014) 6:13. doi: 10.12703/P6-13 24669294 PMC3944738

[30] Mould KJ Jackson ND Henson PM Seibold M Janssen WJ. Single cell RNA sequencing identifies unique inflammatory airspace macrophage subsets. JCI Insight (2019) 4(5):e126556. doi: 10.1172/jci.insight.126556 30721157 PMC6483508

[31] Vankrunkelsven A De Ceulaer K Hsu D Liu FT De Baetselier P Stijlemans B. Lack of galectin-3 alleviates trypanosomiasis-associated anemia of inflammation. Immunobiology (2010) 215:833–41. doi: 10.1016/j.imbio.2010.05.028 20605052

[32] Stijlemans B Brys L Korf H Bieniasz-Krzywiec P Sparkes A Vansintjan L. MIF-mediated hemodilution promotes pathogenic anemia in experimental African trypanosomosis. PloS Pathog (2016) 12:e1005862. doi: 10.1371/journal.ppat.1005862 27632207 PMC5025191

[33] Magez S Truyens C Merimi M Radwanska M Stijlemans B Brouckaert P. P75 tumor necrosis factor-receptor shedding occurs as a protective host response during African trypanosomiasis. J Infect Dis (2004) 189:527–39. doi: 10.1086/381151 14745712

[34] Cnops J De Trez C Stijlemans B Keirsse J Kauffmann F Barkhuizen M. NK-, NKT- and CD8-derived IFNγ drives myeloid cell activation and erythrophagocytosis, resulting in trypanosomosis-associated acute anemia. PloS Pathog (2015) 11:e1004964. doi: 10.1371/journal.ppat.1004964 26070118 PMC4466398

[35] Cenariu D Iluta S Zimta AA Petrushev B Qian L Dirzu N. Extramedullary hematopoiesis of the liver and spleen. J Clin Med (2021) 10(24):5831. doi: 10.3390/jcm10245831 34945127 PMC8707658

[36] Liao C Prabhu KS Paulson RF. Monocyte-derived macrophages expand the murine stress erythropoietic niche during the recovery from anemia. Blood (2018) 132:2580–93. doi: 10.1182/blood-2018-06-856831 PMC6293871 30322871

[37] Mumau MD Vanderbeck AN Lynch ED Golec SB Emerson SG Punt JA. Identification of a multipotent progenitor population in the spleen that is regulated by NR4A1. J Immunol (2018) 200:1078–87. doi: 10.4049/jimmunol.1701250 PMC5793861 29282309

[38] Moras M Lefevre SD Ostuni MA. From erythroblasts to mature red blood cells: Organelle clearance in mammals. Front Physiol (2017) 8:1076. doi: 10.3389/fphys.2017.01076 29311991 PMC5742207

[39] Stijlemans B Leng L Brys L Sparkes A Vansintjan L Caljon G. MIF contributes to *Trypanosoma brucei* associated immunopathogenicity development. PloS Pathog (2014) 10:e1004414. doi: 10.1371/journal.ppat.1004414 25255103 PMC4177988

[40] Claes F Radwanska M Urakawa T Majiwa PA Goddeeris B Büscher P. Variable surface glycoprotein RoTat 1.2 PCR as a specific diagnostic tool for the detection of *Trypanosoma evansi* infections. Kinetoplastid Biol Dis (2004) 3:3. doi: 10.1186/1475-9292-3-3 15377385 PMC521498

[41] Moon S Janssens I Kim KH Stijlemans B Magez S Radwanska M. *Trypanosoma brucei brucei* infection depletes memory b cells resulting in inability of the host to recall protective b cells responses. J Infect Dis (2022) 226 (3):528–40. doi: 10.1093/infdis/jiac112 35363871

[42] Young MD Behjati S. SoupX removes ambient RNA contamination from droplet-based single-cell RNA sequencing data. Gigascience (2020) 9(12):giaa151. doi: 10.1093/gigascience/giaa151 33367645 PMC7763177

[43] Kimmel JC Hwang AB Scaramozza A Marshall WF Brack AS. Aging induces aberrant state transition kinetics in murine muscle stem cells. Development (2020) 147(9):dev183855. doi: 10.1242/dev.183855 32198156 PMC7225128

[44] Stuart T Butler A Hoffman P Hafemeister C Papalexi E Mauck WM 3rd. Comprehensive integration of single-cell data. Cell (2019) 177:1888–1902.e1821. doi: 10.1016/j.cell.2019.05.031 31178118 PMC6687398

[45] Korsunsky I Millard N Fan J Slowikowski K Zhang F Wei K. Fast, sensitive and accurate integration of single-cell data with harmony. Nat Methods (2019) 16:1289–96. doi: 10.1038/s41592-019-0619-0 PMC6884693 31740819

[46] Jin S Guerrero-Juarez CF Zhang L Chang I Ramos R Kuan CH. Inference and analysis of cell-cell communication using CellChat. Nat Commun (2021) 12:1088. doi: 10.1038/s41467-021-21246-9 33597522 PMC7889871

[47] Liberzon A Subramanian A Pinchback R Thorvaldsdóttir H Tamayo P Mesirov JP. Molecular signatures database (MSigDB) 3.0. Bioinformatics (2011) 27:1739–40. doi: 10.1093/bioinformatics/btr260 PMC3106198 21546393

[48] Yu G Wang LG Han Y He QY. clusterProfiler: an r package for comparing biological themes among gene clusters. OMICS (2012) 16:284–7. doi: 10.1089/omi.2011.0118 PMC3339379 22455463

[49] Baral TN De Baetselier P Brombacher F Magez S. Control of *Trypanosoma evansi* infection is IgM mediated and does not require a type I inflammatory response. J Infect Dis (2007) 195:1513–20. doi: 10.1086/515577 17436232

[50] Namangala B De Baetselier P Brijs L Stijlemans B Noël W Pays E. Attenuation of *Trypanosoma brucei* is associated with reduced immunosuppression and concomitant production of Th2 lymphokines. J Infect Dis (2000) 181:1110–20. doi: 10.1086/315322 10720538

[51] Pfefferlé M Ingoglia G Schaer CA Yalamanoglu A Buzzi R Dubach IL. Hemolysis transforms liver macrophages into antiinflammatory erythrophagocytes. J Clin Invest (2020) 130:5576–90. doi: 10.1172/JCI137282 PMC7524492 32663195

[52] Ley K. M1 means kill; M2 means heal. J Immunol (2017) 199:2191–3. doi: 10.4049/jimmunol.1701135 28923980

[53] Stijlemans B Beschin A Magez S Van Ginderachter JA De Baetselier P. Iron homeostasis and *Trypanosoma brucei* associated immunopathogenicity development: A Battle/Quest for iron. BioMed Res Int (2015) 2015:819389. doi: 10.1155/2015/819389 26090446 PMC4450282

[54] Dutta P Hoyer FF Grigoryeva LS Sager HB Leuschner F Courties G. Macrophages retain hematopoietic stem cells in the spleen *via* VCAM-1. J Exp Med (2015) 212:497–512. doi: 10.1084/jem.20141642 25800955 PMC4387283

[55] Hewitt KJ Johnson KD Gao X Keles S Bresnick EH. The hematopoietic stem and progenitor cell cistrome: GATA factor-dependent cis-regulatory mechanisms. Curr Top Dev Biol (2016) 118:45–76. doi: 10.1016/bs.ctdb.2016.01.002 27137654 PMC8572122

[56] Tallack MR Whitington T Yuen WS Wainwright EN Keys JR Gardiner BB. A global role for KLF1 in erythropoiesis revealed by ChIP-seq in primary erythroid cells. Genome Res (2010) 20:1052–63. doi: 10.1101/gr.106575.110 PMC2909569 20508144

[57] Hu T Ghazaryan S Sy C Wiedmeyer C Chang V Wu L. Concomitant inactivation of Rb and E2f8 in hematopoietic stem cells synergizes to induce severe anemia. Blood (2012) 119:4532–42. doi: 10.1182/blood-2011-10-388231 PMC3362366 22422820

[58] Anguita E Candel FJ Chaparro A Roldán-Etcheverry JJ. Transcription factor GFI1B in health and disease. Front Oncol (2017) 7:54. doi: 10.3389/fonc.2017.00054 28401061 PMC5368270

[59] Kadri Z Lefevre C Goupille O Penglong T Granger-Locatelli M Fucharoen S. Erythropoietin and IGF-1 signaling synchronize cell proliferation and maturation during erythropoiesis. Genes Dev (2015) 29:2603–16. doi: 10.1101/gad.267633.115 PMC4699388 26680303

[60] Wang Y Huang Y Yang J Zhou FQ Zhao L Zhou H. Pyruvate is a prospective alkalizer to correct hypoxic lactic acidosis. Mil Med Res (2018) 5:13. doi: 10.1186/s40779-018-0160-y 29695298 PMC5918562

[61] Pan W Ogunremi O Wei G Shi M Tabel H. CR3 (CD11b/CD18) is the major macrophage receptor for IgM antibody-mediated phagocytosis of African trypanosomes: diverse effect on subsequent synthesis of tumor necrosis factor alpha and nitric oxide. Microbes Infect (2006) 8:1209–18. doi: 10.1016/j.micinf.2005.11.009 16616573

[62] Liu G Fu Y Yosri M Chen Y Sun P Xu J. CRIg plays an essential role in intravascular clearance of bloodborne parasites by interacting with complement. Proc Natl Acad Sci (2019) 116:24214–20. doi: 10.1073/pnas.1913443116 PMC6883839 31723045

[63] Lopez R Demick KP Mansfield JM Paulnock DM. Type I IFNs play a role in early resistance, but subsequent susceptibility, to the African trypanosomes. J Immunol (2008) 181:4908–17. doi: 10.4049/jimmunol.181.7.4908 PMC2582636 18802094

[64] Rojas F Matthews KR. Quorum sensing in African trypanosomes. Curr Opin Microbiol (2019) 52:124–9. doi: 10.1016/j.mib.2019.07.001 31442903

[65] Rojas F Silvester E Young J Milne R Tettey M Houston DR. Oligopeptide signaling through TbGPR89 drives trypanosome quorum sensing. Cell (2019) 176:306–317.e316. doi: 10.1016/j.cell.2018.10.041 30503212 PMC6333907

[66] Shi M Pan W Tabel H. Experimental African trypanosomiasis: IFN-gamma mediates early mortality. Eur J Immunol (2003) 33:108–18. doi: 10.1002/immu.200390013 12594839

[67] Doyle AG Herbein G Montaner LJ Minty AJ Caput D Ferrara P. Interleukin-13 alters the activation state of murine macrophages *in vitro*: comparison with interleukin-4 and interferon-gamma. Eur J Immunol (1994) 24:1441–5. doi: 10.1002/eji.1830240630 7911424

[68] Mills CD Kincaid K Alt JM Heilman MJ Hill AM. M-1/M-2 macrophages and the Th1/Th2 paradigm. J Immunol (2000) 164:6166–73. doi: 10.4049/jimmunol.164.12.6166 10843666

[69] Gómez-Rodríguez J Stijlemans B De Muylder G Korf H Brys L Berberof M. Identification of a parasitic immunomodulatory protein triggering the development of suppressive M1 macrophages during African trypanosomiasis. J Infect Dis (2009) 200:1849–60. doi: 10.1086/648374 19911988

[70] Raes G Noël W Beschin A Brys L De Baetselier P Hassanzadeh GH. FIZZ1 and ym as tools to discriminate between differentially activated macrophages. Dev Immunol (2002) 9:151–9. doi: 10.1080/1044667031000137629 PMC2276098 12892049

[71] Stijlemans B Vankrunkelsven A Caljon G Bockstal V Guilliams M Bosschaerts T. The central role of macrophages in trypanosomiasis-associated anemia: rationale for therapeutical approaches. Endocr Metab Immune Disord Drug Targets (2010) 10:71–82. doi: 10.2174/187153010790827966 20158497

[72] Xue J Schmidt SV Sander J Draffehn A Krebs W Quester I. Transcriptome-based network analysis reveals a spectrum model of human macrophage activation. Immunity (2014) 40:274–88. doi: 10.1016/j.immuni.2014.01.006 PMC3991396 24530056

[73] Cuervo H Guerrero NA Carbajosa S Beschin A De Baetselier P Gironès N. Myeloid-derived suppressor cells infiltrate the heart in acute *Trypanosoma cruzi* infection. J Immunol (2011) 187:2656–65. doi: 10.4049/jimmunol.1002928 21804013

[74] Mattila JT Ojo OO Kepka-Lenhart D Marino S Kim JH Eum SY. Microenvironments in tuberculous granulomas are delineated by distinct populations of macrophage subsets and expression of nitric oxide synthase and arginase isoforms. J Immunol (2013) 191:773–84. doi: 10.4049/jimmunol.1300113 PMC3746594 23749634

[75] Maloney J Keselman A Li E Singer SM. Macrophages expressing arginase 1 and nitric oxide synthase 2 accumulate in the small intestine during giardia lamblia infection. Microbes Infect (2015) 17:462–7. doi: 10.1016/j.micinf.2015.03.006 PMC4461514 25797399

[76] Blériot C Chakarov S Ginhoux F. Determinants of resident tissue macrophage identity and function. Immunity (2020) 52:957–70. doi: 10.1016/j.immuni.2020.05.014 32553181

[77] Nobs SP Kopf M. Tissue-resident macrophages: guardians of organ homeostasis. Trends Immunol (2021) 42:495–507. doi: 10.1016/j.it.2021.04.007 33972166

[78] Liu G Abas O Strickland AB Chen Y Shi M. CXCR6+CD4+ T cells promote mortality during *Trypanosoma brucei* infection. PloS Pathog (2021) 17:e1009968. doi: 10.1371/journal.ppat.1009968 34614031 PMC8523071

[79] Kariuki CK Stijlemans B Magez S. The trypanosomal transferrin receptor of *Trypanosoma brucei*-a review. Trop Med Infect Dis (2019) 4(4):126. doi: 10.3390/tropicalmed4040126 31581506 PMC6958415

[80] Kim A Nemeth E. New insights into iron regulation and erythropoiesis. Curr Opin Hematol (2015) 22:199–205. doi: 10.1097/MOH.0000000000000132 25710710 PMC4509743

[81] Paulson RF Ruan B Hao S Chen Y. Stress erythropoiesis is a key inflammatory response. Cells (2020) 9. doi: 10.3390/cells9030634 PMC7140438 32155728

[82] Bennett LF Liao C Quickel MD Yeoh BS Vijay-Kumar M Hankey-Giblin P. Inflammation induces stress erythropoiesis through heme-dependent activation of SPI-c. Sci Signal (2019) 12. doi: 10.1126/scisignal.aap7336 PMC6904953 31506384

[83] Zhang J Wu K Xiao X Liao J Hu Q Chen H. Autophagy as a regulatory component of erythropoiesis. Int J Mol Sci (2015) 16:4083–94. doi: 10.3390/ijms16024083 PMC4346945 25689426

[84] Mei Y Liu Y Ji P. Understanding terminal erythropoiesis: An update on chromatin condensation, enucleation, and reticulocyte maturation. Blood Rev (2021) 46:100740. doi: 10.1016/j.blre.2020.100740 32798012

[85] Buza JJ Logan-Henfrey L Andrianarivo AG Williams DJ. Rise in erythropoietin concentrations in experimental *Trypanosoma congolense* infection of calves. J Comp Pathol (1995) 113:343–56. doi: 10.1016/S0021-9975(05)80120-3 8746957

[86] Suliman HB Logan-Henfrey L Majiwa PA Ole-Moiyoi O Feldman BF. Analysis of erythropoietin and erythropoietin receptor genes expression in cattle during acute infection with *Trypanosoma congolense* . Exp Hematol (1999) 27:37–45. doi: 10.1016/S0301-472X(98)00019-8 9923442

[87] Naessens J Kitani H Nakamura Y Yagi Y Sekikawa K Iraqi F. TNF-alpha mediates the development of anaemia in a murine *Trypanosoma brucei rhodesiense* infection, but not the anaemia associated with a murine *Trypanosoma congolense* infection. Clin Exp Immunol (2005) 139:405–10. doi: 10.1111/j.1365-2249.2004.02717.x PMC1809320 15730385

[88] Rhodes CE Varacallo M. Physiology, Oxygen Transport. In: StatPearls. Treasure Island (FL): StatPearls Publishing (2021). 30855920

[89] Landau D London L Bandach I Segev Y. The hypoxia inducible factor/erythropoietin (EPO)/EPO receptor pathway is disturbed in a rat model of chronic kidney disease related anemia. PloS One (2018) 13:e0196684. doi: 10.1371/journal.pone.0196684 29738538 PMC5940200

[90] Wang H Liu D Song P Jiang F Chi X Zhang T. Exposure to hypoxia causes stress erythropoiesis and downregulates immune response genes in spleen of mice. BMC Genomics (2021) 22:413. doi: 10.1186/s12864-021-07731-x 34090336 PMC8178839

[91] Finch CA Gollnick PD Hlastala MP Miller LR Dillmann E Mackler B. Lactic acidosis as a result of iron deficiency. J Clin Invest (1979) 64:129–37. doi: 10.1172/JCI109431 PMC372098 447849

[92] Haji-Michael PG Ladrière L Sener A Vincent JL Malaisse WJ. Leukocyte glycolysis and lactate output in animal sepsis and ex vivo human blood. Metabolism (1999) 48:779–85. doi: 10.1016/S0026-0495(99)90179-8 10381154

[93] Richard A Vallin E Romestaing C Roussel D Gandrillon O Gonin-Giraud S. Erythroid differentiation displays a peak of energy consumption concomitant with glycolytic metabolism rearrangements. PloS One (2019) 14:e0221472. doi: 10.1371/journal.pone.0221472 31483850 PMC6726194

[94] Possemiers H Vandermosten L Van Den Steen PE. Etiology of lactic acidosis in malaria. PloS Pathog (2021) 17:e1009122. doi: 10.1371/journal.ppat.1009122 33411818 PMC7790250

[95] Costa Leite T Da Silva D Guimarães Coelho R Zancan P Sola-Penna M. Lactate favours the dissociation of skeletal muscle 6-phosphofructo-1-kinase tetramers down-regulating the enzyme and muscle glycolysis. Biochem J (2007) 408:123–30. doi: 10.1042/BJ20070687 PMC2049071 17666012

[96] Leite TC Coelho RG Da Silva D Coelho WS Marinho-Carvalho MM Sola-Penna M. Lactate downregulates the glycolytic enzymes hexokinase and phosphofructokinase in diverse tissues from mice. FEBS Lett (2011) 585:92–8. doi: 10.1016/j.febslet.2010.11.009 21074528

[97] Ishii H Nishida Y. Effect of lactate accumulation during exercise-induced muscle fatigue on the sensorimotor cortex. J Phys Ther Sci (2013) 25:1637–42. doi: 10.1589/jpts.25.1637 PMC3885857 24409038

[98] Jha MK Song GJ Lee MG Jeoung NH Go Y Harris RA. Metabolic connection of inflammatory pain: pivotal role of a pyruvate dehydrogenase kinase-pyruvate dehydrogenase-lactic acid axis. J Neurosci (2015) 35:14353–69. doi: 10.1523/JNEUROSCI.1910-15.2015 PMC6605420 26490872

[99] Wesson DE Buysse JM Bushinsky DA. Mechanisms of metabolic acidosis-induced kidney injury in chronic kidney disease. J Am Soc Nephrol (2020) 31:469–82. doi: 10.1681/ASN.2019070677 PMC7062220 31988269

[100] Bugarski M Ghazi S Polesel M Martins JR Hall AM. Changes in NAD and lipid metabolism drive acidosis-induced acute kidney injury. J Am Soc Nephrol (2021) 32:342–56. doi: 10.1681/ASN.2020071003 PMC8054907 33478973

[101] Paul M Stefaniak J Smuszkiewicz P Van Esbroeck M Geysen D Clerinx J. Outcome of acute East African trypanosomiasis in a polish traveller treated with pentamidine. BMC Infect Dis (2014) 14:1–8. doi: 10.1186/1471-2334-14-111 24571399 PMC3941560

[102] Darling TN Balber AE Blum JJ. A comparative study of d-lactate, l-lactate and glycerol formation by four species of leishmania and by *Trypanosoma lewisi* and *Trypanosoma brucei gambiense* . Mol Biochem Parasitol (1988) 30:253–7. doi: 10.1016/0166-6851(88)90094-1 3054535

[103] Podolec P Szabó AH Blaško J Kubinec R Górová R Višňovský J. Direct silylation of trypanosoma brucei metabolites in aqueous samples and their GC-MS/MS analysis. J Chromatogr B Analyt Technol BioMed Life Sci (2014) 967:134–8. doi: 10.1016/j.jchromb.2014.07.023 25089958

[104] Uzcátegui NL Figarella K Segnini A Marsiccobetre S Lang F Beitz E. *Trypanosoma brucei* aquaglyceroporins mediate the transport of metabolic end-products: Methylglyoxal, d-lactate, l-lactate and acetate. Biochim Biophys Acta Biomembr (2018) 1860:2252–61. doi: 10.1016/j.bbamem.2018.09.008 30409521

