# Corrigendum: Tipping the balance between erythroid cell differentiation and induction of anemia in response to the inflammatory pathology associated with chronic trypanosome infections

**DOI:** 10.3389/fimmu.2023.1357960

**Published:** 2024-01-09

**Authors:** Hang Thi Thu Nguyen, Magdalena Radwanska, Stefan Magez

**Affiliations:** ^1^ Department of Biochemistry and Microbiology, Ghent University, Ghent, Belgium; ^2^ Laboratory for Biomedical Research, Ghent University Global Campus, Incheon, Republic of Korea; ^3^ Laboratory of Cellular and Molecular Immunology, Vrije Universiteit Brussel, Brussels, Belgium; ^4^ Department of Biomedical Molecular Biology, Ghent University, Ghent, Belgium

**Keywords:** trypanosomosis, tissue resident macrophages, extramedullary erythropoiesis, immunopathology, lactic acidosis

In the published article, there was an error in the Data Availability statement, as two datasets were omitted. The correct Data Availability statement is:

**Data Availability Statement**

scRNA sequencing (scRNA-seq) datasets used in this study are publicly available in the BioStudies EMBL-EBI database (www.ebi.ac.uk/biostudies). The datasets can be accessed under the accession numbers E-MTAB-10174, which originated from https://doi.org/10.1371/journal.ppat.1010026, and E-MTAB-12109.

The authors apologize for this error and state that this does not change the scientific conclusions of the article in any way. The original article has been updated.

