# Supplementary material for: Tipping the balance between erythroid cell differentiation and induction of anemia in response to the inflammatory pathology associated with chronic trypanosome infections
Source: Front Immunol. 2022 Nov 7;13:1051647. doi: 10.3389/fimmu.2022.1051647 (PMC9676970; doi:10.3389/fimmu.2022.1051647)
Supplement: Supplementary file 1 [file DataSheet_1.docx]

Supplementary Material


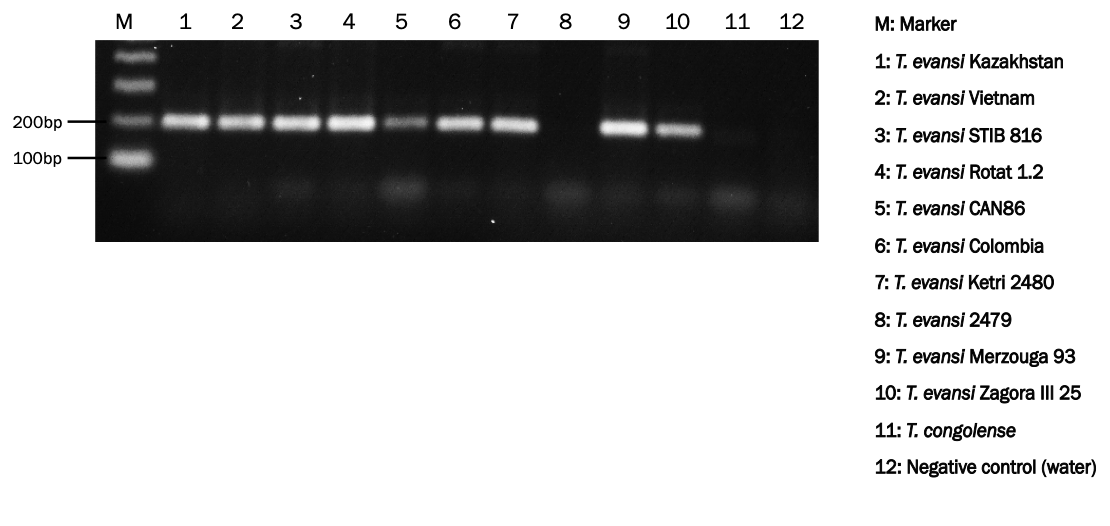


**Figure S1: Detection of Rotat1.2 gene by PCR**

| **Table S1. Rank test for virulence classification** | |  |
| --- | --- | --- |
|  |  |  |
|  | **Logrank test for trend** | **Gehan-Breslow-Wilcoxon test** |
| Chi square | 77.68 | 74.82 |
| df | 1 | 3 |
| P value | <0.0001 | <0.0001 |
| P value summary | **** | **** |

**
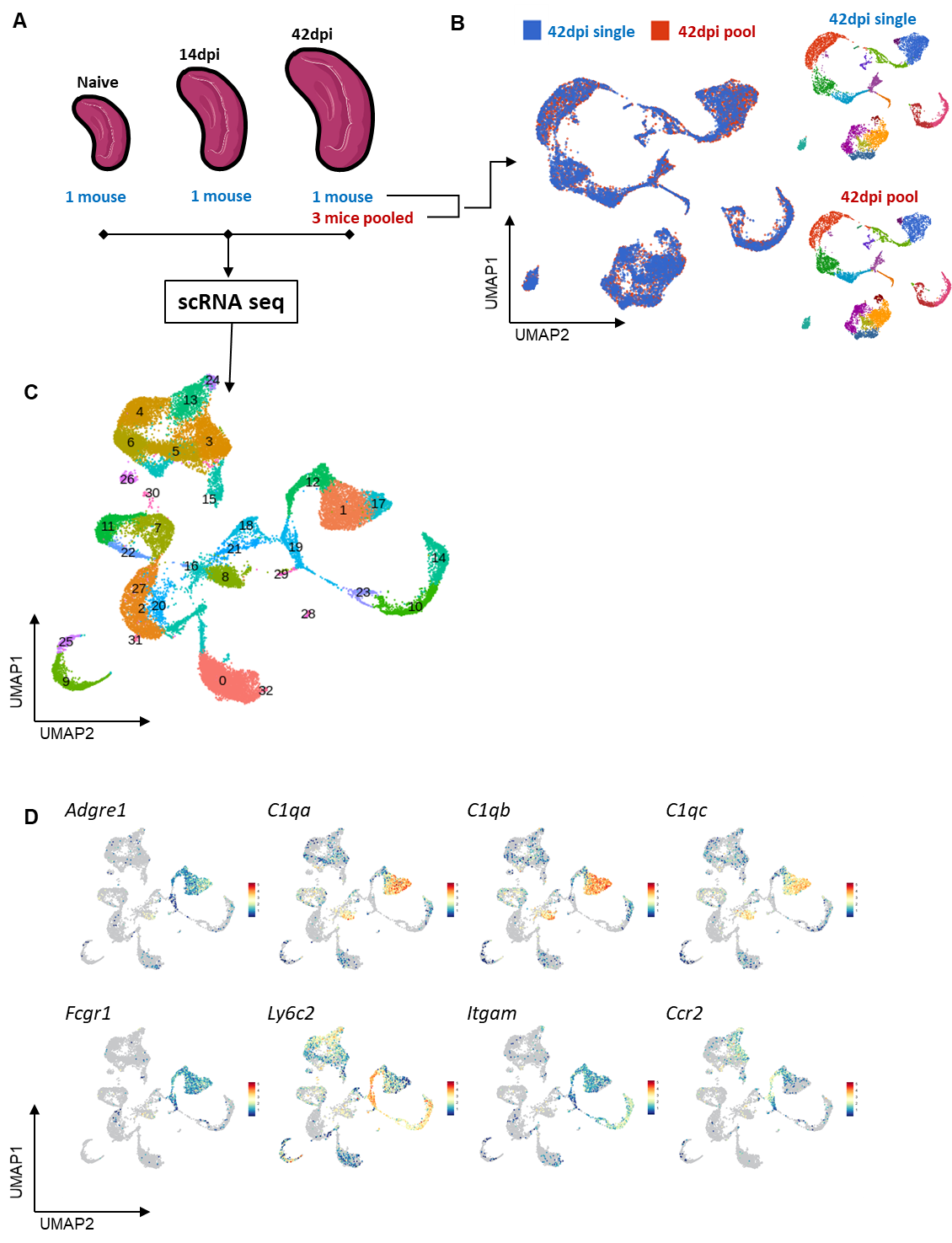
**

**Figure S2:** (**A**) Experimental setup of scRNA-seq experiment. (**B**) UMAP projection of total splenocytes derived from 42dpi single and 42dpi pool datasets colored by sample origin (left panel) or clusters (right panel) showing an exceptional similarity between the two datasets, with homogeneous cell distribution in clusters, indicating experimental reproducibility. (**C**)UMAP showing expression of total spleen cells combined from all datasets. (**D)** Expression of gene markers for monocytes and macrophages population.


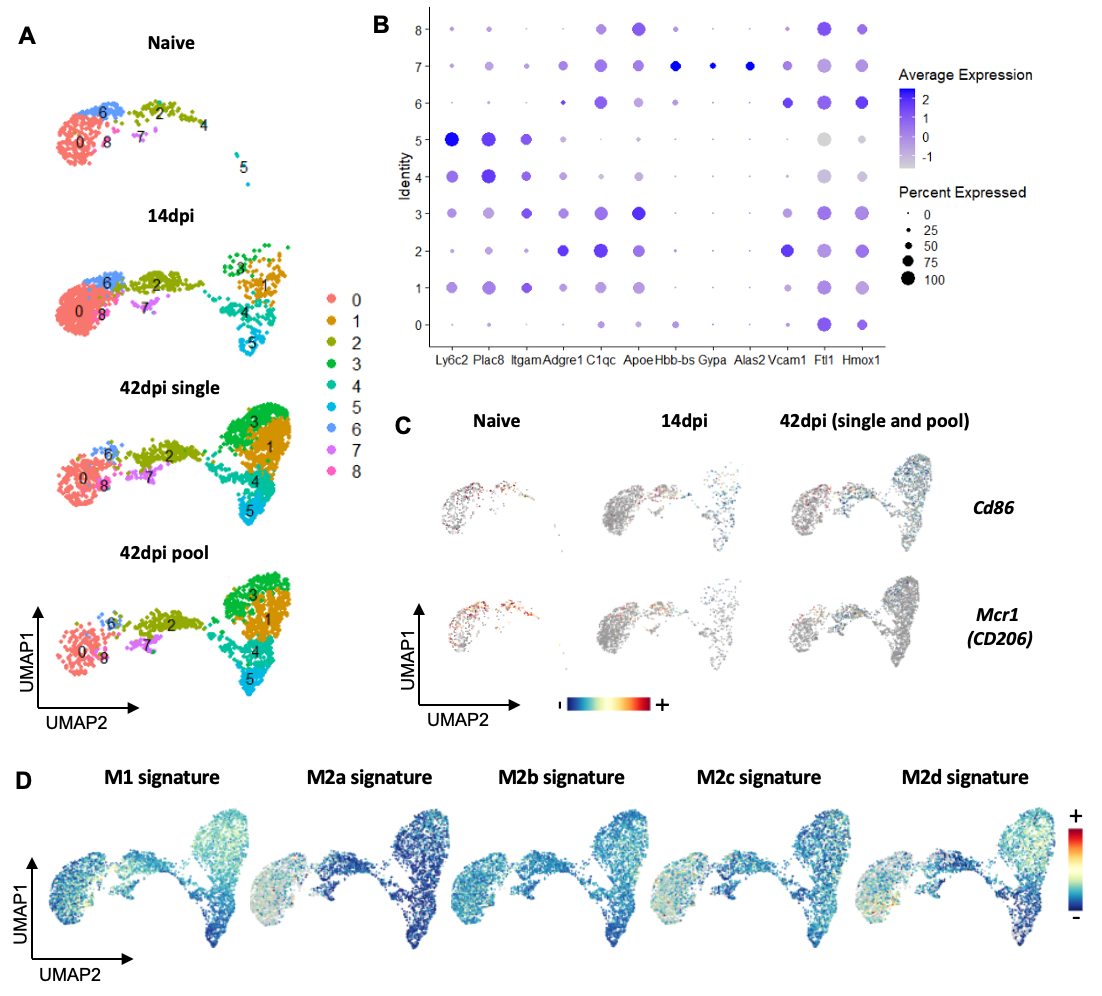


**Figure S3:** (**A**) UMAP projection of Macrophage-only dataset splited by time-points. (**B**) Dotplot showing average expression of gene markers used for Macrophages annotation. (**C**) UMAP showing expression of Cd86 (upper panels) and Mrc1 (CD206) (lower panels) genes at 3 time-point separately. (**D**) Mean expression of combined genes hallmarked for M1 and M2a/M2b/M2c/M2d macrophages signature.


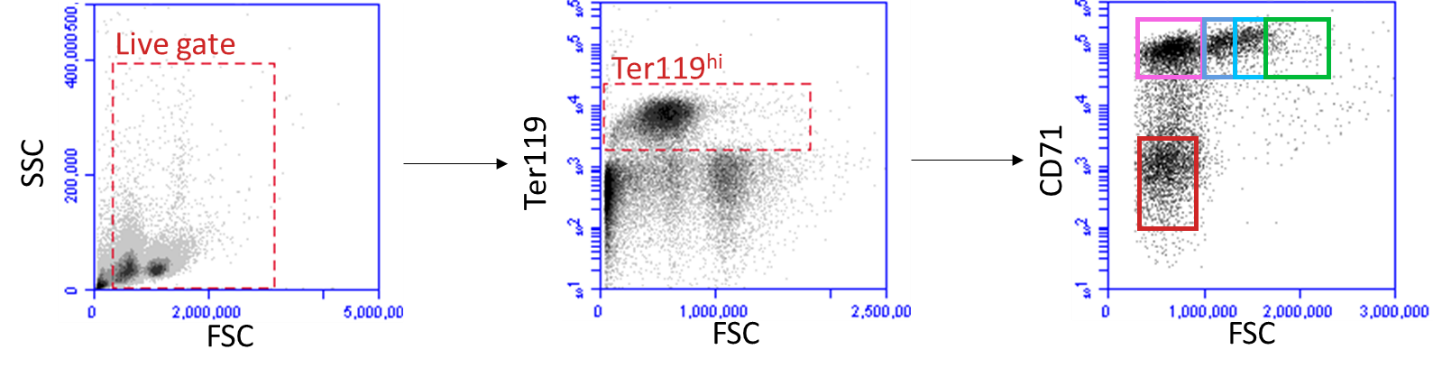
**Figure S4:** **Gating strategy used for erythrocytes lineage analysis by flow cytometry.**
